# Individualization and Purification of Carbon Nanothreads

**DOI:** 10.1002/smll.74445

**Published:** 2026-08-03

**Authors:** George Bepete, Sikai Wu, Bohan Xu, Ámbar E. Escobar Colón, Andres Fest, Da Zhou, Yu Lei, Methembe Moyo, Zhuohang Yu, George Chimowa, Kazunori Fujisawa, Nestor Perea‐Lopez, Vincent H. Crespi, Mauricio Terrones

**Affiliations:** ^1^ Department of Physics The Pennsylvania State University University Park Pennsylvania USA; ^2^ Department of Chemistry The Pennsylvania State University University Park Pennsylvania USA; ^3^ Center for 2‐Dimensional and Layered Materials Centre for Atomically Thin Multifunctional Coatings The Pennsylvania State University University Park Pennsylvania USA; ^4^ Materials Research Institute The Pennsylvania State University University Park Pennsylvania USA; ^5^ Department of Chemical and Materials Engineering Centre for NanoScience Research (CeNSR) Concordia University Montreal Canada; ^6^ Department of Physics Centre for NanoScience Research (CeNSR) Concordia University Montreal Canada; ^7^ Department of Materials Science and Engineering The Pennsylvania State University University Park Pennsylvania USA; ^8^ Institute of Materials Research Shenzhen Key Laboratory of Advanced Layered Materials for Value‐added Applications Center of Double Helix Guangdong Provincial Key Laboratory of Thermal Management Engineering and Materials Tsinghua Shenzhen International Graduate School Shenzhen Guangdong China; ^9^ Department of Physics and Astronomy Botswana International University of Science and Technology Palapye Botswana; ^10^ Research Initiative for Supra‐Materials and Global Aqua Innovation Centre Shinshu University Nagano Japan

**Keywords:** carbon nanothreads, dispersion, individualization, intercalation, potassium, thin films

## Abstract

Notwithstanding the synthesis of many types of carbon nanothreads with different structures, compositions, and theoretical property predictions, the experimental verification of thread properties remains challenging due to a paucity of techniques to disperse as‐grown solid‐state nanothread samples. In this work, we demonstrate that pyridine‐derived carbon nitride nanothread samples can be intercalated with potassium to produce reduced carbon nitride nanothreads that can be readily dispersed in polar organic solvents such as dimethyl sulfoxide. Atomic force microscopy of thin films produced from these dispersions reveals thread‐like objects as narrow as 0.6 nm in height, suggesting exfoliation and dispersion into individualized threads and small bundles. The individualized nanothreads emit blue/green luminescence, suggesting potential utility in optoelectronic applications. Remarkably, the temperature‐dependent conductivity of a thin film of nanothreads shows weakly semiconducting behavior, falling only ∼40% from room temperature to 2 K. The reductive dissolution approach reported in this work greatly improves the processability of as‐grown nanothread materials, thus opening opportunities for single‐nanothread characterization, post‐synthetic functionalization, thin film fabrication, and the production of nanothread composites.

## Graphical abstract

Reductive dissolution enables the solution processing of carbon nitride nanothreads into individualized nanothreads that can be assembled into ultrathin films. The resulting interconnected networks exhibit weakly semiconducting behavior, with the conductivity decreasing by only ∼40% from room temperature to 2 K.

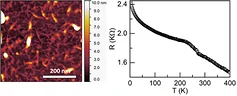

## 1 Introduction

Carbon nanothreads are ultrathin, extended one‐dimensional (1D) frameworks built around a strong sp^3^‐bonded carbon backbone [1]. Theoretical calculations suggest that these materials may possess remarkable tensile strength and thermal conductivity that rivals those of single‐walled carbon nanotubes (SWCNTs), making them attractive candidates for high‐performance composites [2, 3]. In contrast to SWCNTs, nanothreads of diverse compositions and backbone structures are possible by tuning precursor chemistry within a broad class of unsaturated and typically aromatic organic molecules [1, 4, 5, 6, 7, 8, 9, 10, 11, 12, 13, 14, 15, 16, 17]. This enables facile incorporation of heteroatoms and functional groups to tune physical and chemical properties of nanothread materials [18, 19, 20, 21, 22]. For example, compared to benzene‐derived threads, pyridine‐derived nanothreads show a smaller electronic bandgap and stronger light emission at longer wavelengths [23]. The introduction of nitrogen may also improve tensile and bending stiffness [24]. The versatility of nanothread synthesis suggests prospects for multifunctional materials that combine a high‐strength backbone with added functionalities. Nanothread materials are anticipated to find applications in next‐generation high‐performance materials such as nanofibers, optoelectronics, sensing, energy storage, photocatalysis, and other domains [2, 18, 21, 22, 25, 26, 27].

**FIGURE 1:**
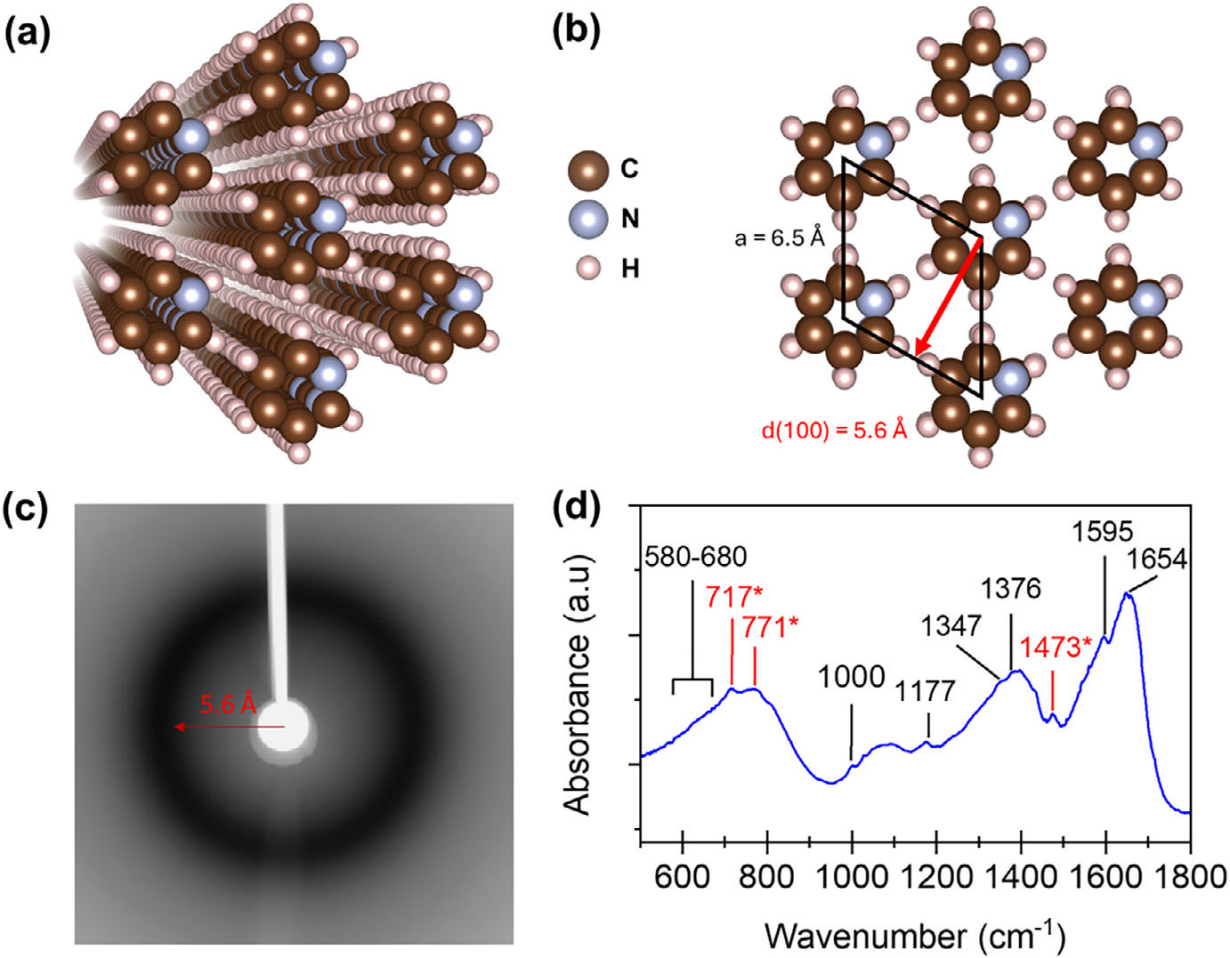
Synthesis of carbon nitride nanothreads. Schematic representation of the bundling in carbon nanothread crystals. (a) 3D view of a bundle of fully saturated carbon nitride nanothreads. (b) Hexagonal packing of the carbon nitride nanothreads viewed down the hexagonal c‐axis. The unit cell parameter and d‐spacing of the (100) plane are labeled. (c) X‐ray diffraction pattern and (d) FTIR spectrum of the as‐synthesized carbon nitride nanothreads recovered from the compression of pyridine in a Paris‐Edinburgh press. The peaks labeled with asterisks may be associated with amorphous byproducts or pyridinic linkers in the resulting nanothreads.

Many potential applications of carbon nanothreads will require manipulation of exfoliated nanothread samples with high weight fractions of individual nanothreads or small bundles dispersed in the liquid phase. However, the solid‐state nature of nanothreads produced by high‐pressure synthesis poses a challenge. Nanothreads are typically formed by compression of multiply unsaturated precursors to 10–30 GPa at room temperature where 1D molecular stacks of precursor crystals polymerize into nanothreads [1, 6, 11, 12, 16, 25, 28]. Because of their rigidity and high aspect ratio, the resulting threads tend to close‐pack into a pseudohexagonal 2D lattice (Figure 1a,b) [1, 29]. The packing of nanothreads is maintained at ambient pressure through van der Waals [1, 3], dipole−dipole interactions, and sometimes hydrogen bonding interactions [14, 30]. Thread rigidity and inter‐thread adhesion makes as‐grown nanothread samples challenging to exfoliate, solvate, and process into, for example, thin films, purified thread samples, single‐nanothread devices, or composites. Developing such processing capabilities is critical for realizing full technological potential of nanothread materials. Conventional methods such as sonication have been applied in the dispersion of SWCNTs, but carbon nanothreads tend to form larger bundles (of thousands of nanothreads [1, 29]) when compared to SWCNT bundles (less than hundred nanotubes) [31, 32, 33], making near‐complete dispersion challenging.

This study provides experimental evidence that pyridine‐derived carbon nitride nanothreads can be dispersed as individualized nanothreads in organic solvents, through reduction(intercalation) with potassium (K). The K‐intercalated as‐synthesized nanothread sample readily dispersed in a polar aprotic solvent, such as dimethyl sulfoxide (DMSO) and *N*‐methyl‐2‐pyrrolidone (NMP), to form a stable suspension of individualized carbon nitride nanothreads. Optical spectroscopy of the dispersion shows absorption from 300 to 450 nm and a broad photoluminescence (PL) peak between 350 and 650 nm. Atomic force microscopy (AFM) of carbon nitride nanothread thin films shows a network of small bundles and individual nanothreads as thin as 0.6 nm. Fourier transform infrared (FTIR) spectroscopy and X‐ray photoelectron spectroscopy (XPS) suggest that our processing method did not introduce unwanted chemical modifications to the carbon nitride nanothread structures, but rather aided in purifying the samples by removing amorphous carbon nitride content. The thin films exhibit weakly semiconducting behavior in the temperature‐dependent conductivity.

## 2 Results and Discussion

### 2.1 Nanothread Synthesis From Pyridine

Slow compression and decompression of pyridine as reported by Li et al. was carried out in a Paris‐Edinburgh (PE) press to prepare solid‐state samples of carbon nitride nanothreads of nominal composition (C_5_H_5_N)_n_ [5]. To confirm the formation of nanothreads, the recovered product was characterized by X‐ray diffraction (XRD), FTIR, and transmission electron microscopy (TEM). XRD of the as‐synthesized sample exhibits a broad diffraction ring at a d‐spacing of approximately 5.6 Å (Figure 1c and Figure S1, Supporting Information). This characteristic d‐spacing is associated with the 2D pseudohexagonal packing (bundling) of carbon nitride nanothreads as illustrated in the schemes of Figure 1a,b [5]. The broadness of the diffraction peak reflects limited long‐range nanothread packing order, which may result from less pronounced uniaxial stress in PE press than in the diamond anvil cell (DAC) [5, 34, 35, 36]. Disruptions of long‐range nanothread packing order may also occur due to structural relaxation of nanothreads, melting or evaporation of unreacted precursor, and mechanical stress upon decompression and sample recovery [11, 35].

The FTIR spectrum of the as‐synthesized sample (Figure 1d) shows an overall profile similar to that observed in the previous synthesis of carbon nitride nanothreads [5], although broader IR peaks indicate a less uniform local chemical environment. The sp^3^ C─H stretching mode (2850–2960 cm^−1^) in Figure S2 (Supporting Information) and C═C/C═N stretching modes (1585–1680 cm^−1^) are observed, as expected for carbon nitride nanothreads with fully or partially saturated backbones [5]. The two diagnostic vibrational modes characteristic of carbon nitride nanothreads are also observed: radial breathing modes at 580–680 cm^−1^ and a mode around 1117 cm^−1^, which is associated with axial elongation and compression of the C─N bonds between C_5_H_5_N rings (inter‐ring C─N stretching) [5]. The relatively low intensities and broadening of these modes may be due to reduced uniformities in nanothread packing and their atomic structures in the as‐synthesized sample. Like the previously synthesized carbon nitride nanothread product in a diamond anvil cell, the FTIR spectrum (Figure 1d) also reveals the presence of amorphous carbon nitride content in the as‐synthesized sample. The peaks marked by asterisks * at 717, 771, and 1473 cm^−1^ are commonly associated with amorphous carbon nitride [5, 37, 38]. The broad, intense O−H/N−H stretching modes at above 3000 cm^−1^ (Figure S2, Supporting Information) may be attributed to moisture absorbed from air after synthesis or the presence of byproducts from side reactions (such as transfer hydrogenation) under pressure. Although sp^2^ C–H stretching mode (3000–3100 cm^−1^) is also expected in this region, it is likely obscured by these broad/intense O–H/N–H features.

To further inspect the sample morphology, TEM specimens were prepared by drop‐casting method after the as‐synthesized sample was sonicated in ethanol. Figure S3a (Supporting Information) shows overlapping domains with 1D fringe‐like contrast alongside regions that are less ordered or amorphous‐like. The fringe‐like contrast degraded drastically within seconds even under gentle electron‐dose conditions (Figure S3b, Supporting Information), a behavior expected for strained, 1D covalent frameworks. Together, the XRD, FTIR, and TEM results support the formation of nanothreads in the as‐synthesized sample, despite the limited crystallinity in nanothread packing and the presence of some amorphous carbon nitride content. The crystallinity of products recovered from nanothread synthesis can be affected by many factors, such as uniaxial stress conditions, recovery yield, structural relaxation, and reaction selectivity in nanothread formation pathways [5, 11, 14, 16, 17, 35, 36]. Regardless of the degree of sample crystallinity, a method to extract and disperse nanothreads from these solid‐state products is essential for enabling further investigations and applications of nanothread materials.

The reductive dissolution reported here to disperse and individualize carbon nitride nanothreads requires modest heat treatment (< 200°C). To assess the thermal stability of the as‐synthesized sample, thermogravimetric analysis (TGA) was performed from 20 to 700°C under argon flow. Figure S4 (Supporting Information) shows that the sample loses about 10% of its mass gradually as it is heated to 300°C and then decomposes at around 400°C. The gradual mass loss from room temperature to 300°C may be attributed to desorption of moisture and unreacted pyridine molecules as well as the decomposition of short oligomers and/or weakly bound amorphous content. TGA analysis suggests that heat treatment up to at least 300°C can be safely conducted during the dispersion and purification of carbon nitride nanothread samples without substantially compromising the nanothreads’ structural integrity.

### 2.2 Reductive Dissolution of Carbon Nitride Nanothreads

As‐synthesized carbon nitride nanothreads were exfoliated and dispersed by reductive dissolution. First, the nanothreads were reduced by mixing 9 mg of the as‐synthesized nanothread sample with 1 mg of potassium metal under an argon atmosphere at 150°C. This experimental potassium‐to‐carbon ratio was chosen so that the intercalation reaction yields an approximate composition of KC_22_, representing a relatively high degree of reduction given that not all carbon atoms are charge‐transfer active in mixed sp^2^/sp^3^‐hybridized nanothread backbones. This stoichiometry is comparable to that of alkali‐intercalated carbons such as KC_8_‐KC_24_ and closely parallels the conditions used to intercalate graphite [39, 40, 41, 42], intercalate anthracite coal [43], and to dope SWCNTs (KC_25_) [44]. The reaction between the dark orange carbon nitride nanothread sample and the shiny potassium metal yielded a dark brown material as a putative potassium salt of carbon nitride nanothreads. Afterward, the sample was allowed to cool to room temperature. The resulting material was either directly used or stored in a tightly sealed glass vial. A similar reduction was also performed on a furan‐derived carbon nanothread sample for XRD characterization.

Due to the bundled nature of carbon nanothreads, potassium metal is expected to infiltrate the sample and be confined within the van der Waals spacings between adjacent nanothreads and within the interstitial channels between nanothreads. Figure S5 (Supporting Information) presents XRD profiles for a sample of furan nanothreads before and after reaction with K. In the pristine nanothreads sample, the diffraction peak at 17.6° corresponds to a d‐spacing of 5.0 Å, consistent with the crystalline packing of furan‐derived nanothreads [11]. After reduction of the nanothreads with K the diffraction peak disappears, indicating the loss of 2D pseudohexagonal nanothread packing, likely due to the intercalation of potassium in between packed nanothreads and the structural relaxation of threads. A similar loss of crystalline order in XRD has been observed for SWCNT bundles upon alkali metal doping [45, 46]. The intercalation of K into purely sp^2^ carbon systems such as graphite or SWCNT bundles is accompanied by charge transfer due to the difference in the electronegativities of potassium and sp^2^ carbon [47]. In carbon nitride nanothreads of mixed sp^2^/sp^3^ character we anticipate that charge transfer likely occurs in proximity to the sp^2^ sections of partially saturated nanothreads [47, 48].

### 2.3 Mechanism of Potassium Intercalation and Reduction in Carbon Nitride Nanothreads

**FIGURE 2:**
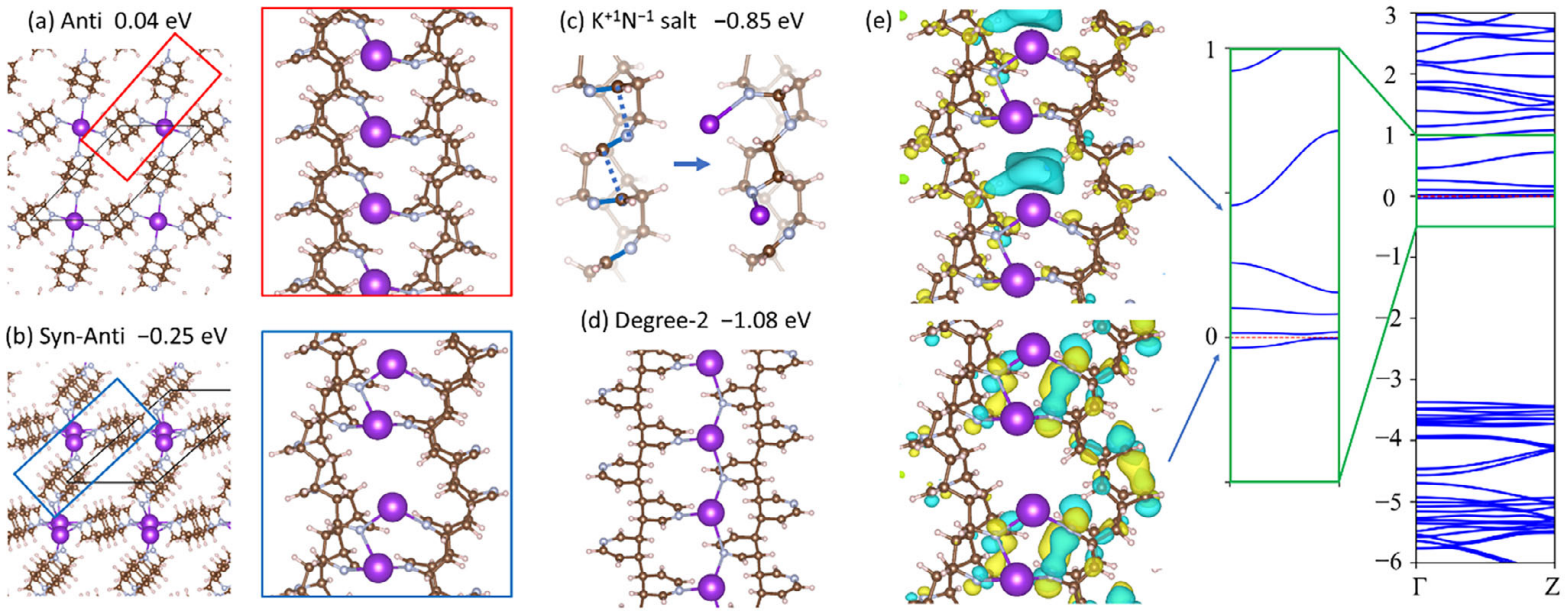
Thread‐potassium interaction energies. Negative values mean intercalation is favorable. (a) Anti thread's C=N π* orbital energy is lowered by the electronegative nitrogen, which allows it to accept electrons from potassium. (b) Syn‐anti thread's C=N π* orbital energy is further lowered by the proximity of adjacent double bonds, which is significantly more favorable for intercalation. (c) The proximity of adjacent double bonds in some cases allows further polymerization induced by potassium and results in –N–^−1^ anions balanced by K^+^ cations. (d) Non‐rigid degree 2 threads can optimize coordination. The π* energy is further lowered by increased π conjugation. (e) The states in the zoomed‐in band structure of a syn‐anti thread are mostly π* orbitals and those resembling potassium s distorted by spatial confinement. One π* orbital is occupied in this example.

We use density functional theory (DFT) to provide mechanistic insights into alkali‐metal intercalation and reduction of nanothreads. Total energies were computed for several representative nanothread backbone motifs and packing arrangements, their potassium‐intercalated counterparts, and bulk potassium using the PBE functional [49], DFT‐D3(BJ) Van der Waals correction [50, 51], and a plane wave basis set with a cutoff of 550 eV as implemented in the VASP software package [52, 53, 54, 55, 56]. The energy per intercalated potassium atom is compared to unintercalated bulk nanothreads and solid potassium at zero temperature (Tables S1–S3, Supporting Information). It is found that potassium can donate electrons to C═N π* orbitals, whose energy is lowered from that of C═C orbitals due to electronegative nitrogen in the double bond. Depending on the thread type, the binding energy per potassium ranges from 0 to −1 eV, where negative values denote an energetic preference for intercalation. The more prevalent π bonding in degree 2 threads (i.e. those in which each monomer precursor makes two bonds to neighbors) yields the most favorable binding energies, because of a smaller HOMO−LUMO gap and lower π* orbital energy. The flexibility of degree 2 threads also affords a more favorable coordination geometry, as shown in Figure 2d. When two or more double bonds along the axis of a degree 4 thread are held in close proximity by the rigid thread backbone, the overlap of non‐bonding π orbitals splits their energy levels. The lowered level facilitates electron acceptance, resulting in a potassium binding energy of about −0.25 eV. One such example is the *syn‐anti* thread of Figure 2b. Threads with C═N π* orbitals that do not utilize these two factors typically have potassium binding energies within of approximately ±0.04 eV of zero, such as the *anti*‐thread of Figure 2a; (*anti* means that adjacent C═N bonds fall on opposite sides of a thread; *syn* means they fall on the same side). In some structures with closely spaced double bonds along the thread axis, the introduction of potassium leads to barrierless reaction of adjacent double bonds during structural relaxation, forming a nitrogen anion with negative one formal charge and a bond order of 2, balanced by a K^+^ cation (Figure 2c). The energy drops by ∼0.85 eV per K when potassium is added to systems of this sort and increases by ∼0.83 eV per K if the potassium is subsequently removed.

We also investigated other potential intercalation mechanisms, including electron donation into a C═C π* orbital and potassium coordination with the N or C─H of the saturated thread backbone. Among these possibilities, the K−N(sp^3^) coordination offered the most competitive binding energy; however, it was still not favorable enough to support the intercalation of sparsely distributed potassium atoms in terms of binding energy. It is important to note that these simulations of sparse intercalation do not rule out other possibilities. For instance, a favorable entropy of mixing could still drive the formation of a potassium liquid–saturated thread mixture. Alternatively, more complex K−K coordination might also stabilize the intercalation with fully saturation thread.

**FIGURE 3:**
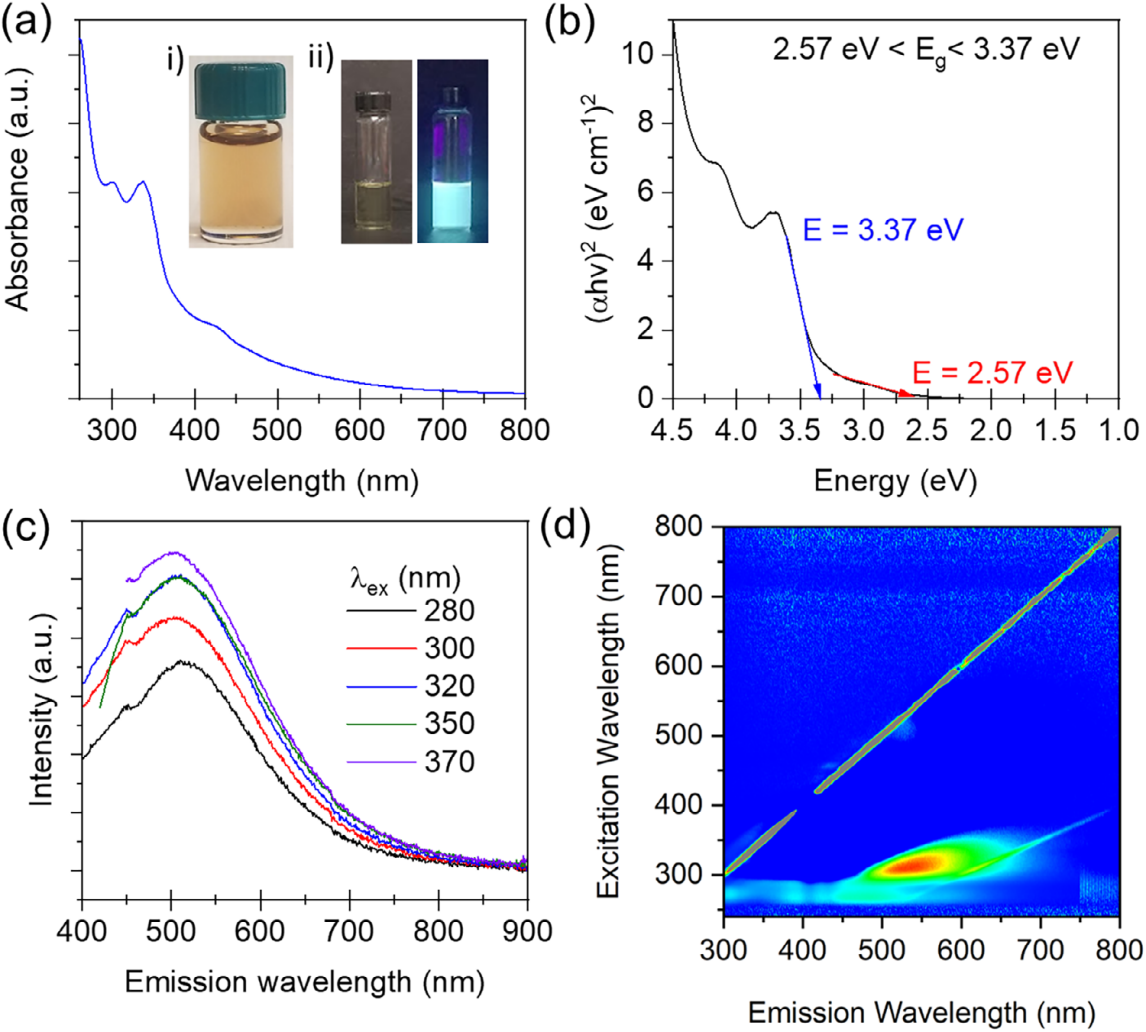
Spectroscopic characterization of suspensions of individualized carbon nitride nanothreads. (a) UV–vis absorption spectrum of carbon nitride nanothread dispersion in DMSO showing absorption peaks at 300, 337, and 420 nm. Inset: (i) A brown solution/dispersion formed when the potassium salt of nanothreads was exposed to DMSO. (ii) A dilute dispersion of nanothreads in DMSO is transparent under normal light and displays strong blue/green fluorescence under 365 nm UV illumination. (b) Tauc plot calculated using an absorption coefficient α and photon energy hν to determine the optical bandgap of the dispersed carbon nitride nanothreads in DMSO, with extrapolation of the linear regions (blue and red lines) estimating an optical bandgap of between 2.57 and 3.37 eV. (c,d) Excitation wavelength‐dependent photoluminescence spectra/map of carbon nanothread dispersions showing a broad peak in the range of 350–650 nm.

In the context of our samples, the XPS N1s spectra (Figure 6c,d) show a comparable proportion of C═N and C─N bonding, and the emission spectra (Figure 3c,d) confirm the presence of emissive sp^2^ segments (*vide infra*). Combined with our theoretical findings, it is suggested that in these mixed sp^2^/sp^3^‐hybridized nanothreads, intercalation is preferred and likely initiated at the sp^2^ regions. The process involves charge donation from potassium to the C═N π* orbital, which facilitates the infiltration of potassium clusters and, in turn, separates the adjacent sp^3^ sections of the threads. While the question of whether pure sp^3^ threads could support potassium intercalation, perhaps through more complex K─K coordination or entropy of mixing, remains open, the abundance of C═N bonding present in the as‐synthesized sample clearly facilitate the intercalation process. The intercalated system can be thought of as a polyelectrolyte that is soluble in organic polar aprotic solvents, akin to the behavior of SWCNT bundles, graphite, and anthracite coal [43, 44, 57, 58, 59].

### 2.4 Exfoliation and Dispersion of K‐Intercalated Nanothreads

A dispersion of carbon nitride nanothreads was obtained by exposing the reduced sample to DMSO for 24 h with gentle stirring and no sonication; sedimentation and centrifugation were then performed to remove large aggregates and oligomers from the dispersion (see Experimental Section). Figure 3a, insert (i) shows the resulting brown dispersion of carbon nitride nanothreads in DMSO. The reduced nanothread sample can also be readily dispersed in other polar aprotic solvents such as tetrahydrofuran (THF) and *N*‐methly‐2‐pyrrolidone (NMP) (Figure S7, Supporting Information).

The optical properties of dispersed threads were examined by absorption and photoluminescence spectroscopy. The UV‐vis absorption spectrum of the dispersion (Figure 3a) shows peaks at 300, 337, and 420 nm. The diluted dispersion of carbon nitride nanothreads in DMSO is colorless and displays bright blue/green fluorescence when illuminated with a 365 nm UV lamp, as shown in insert (ii) of Figure 3a. As the solvent used here (DMSO) is non‐emissive at visible wavelengths, the bright blue luminescence emission must come from the dispersed component of the sample. According to the Tauc plot in Figure 3b, the optical bandgap of the dispersed material falls between 2.6 and 3.4 eV. This range aligns reasonably well with the predicted bandgap for carbon nitride nanothreads, which ranges from 3.09 to 3.48 eV for fully saturated threads and from 1.92 to 3.82 eV for partially saturated threads [23]. The excitation wavelength‐dependent photoluminescence spectra/map of the dispersions in Figure 3c,d show a broad emission from 350 to 650 nm, which may be attributed to bandgap emission. This optical response indicates potential utility in optoelectronic applications [60, 61].

To demonstrate the generality of the exfoliation approach, the same treatment was applied to furan‐derived carbon nanothreads. The UV‐vis absorption spectra of the resulting dispersions (Figures S6 and S8, Supporting Information) show that furan‐derived nanothreads can also be effectively exfoliated and dispersed in DMSO, yielding a dispersion with distinct optical signatures. The carbon nitride nanothreads exhibit a red‐shifted absorption edge relative to the furan‐derived nanothreads, consistent with their smaller predicted band gap (Figure S8, Supporting Information) [5, 23]. These results demonstrate that our exfoliation method is applicable to nanothreads derived from other precursors, including furan, which allow access to tunable electronic properties of nanothread materials through heteroatom chemistry [7, 11, 23, 61, 62].

For easier handling and improved processability, the dispersed carbon nitride nanothreads were precipitated by ultracentrifugation at 12000 rpm and redispersed in water. By this time when the carbon nanothreads were exposed to water, they carried negligible residual charge, and no further reaction was expected. This solvent exchange from DMSO to water provides a clean, low–boiling‐point medium that facilitates handling and is compatible with downstream solution‐processing methods. In addition, exposure of the dispersed carbon nitride nanothreads to air and water leads to the reaction of residual potassium with oxygen and water, forming potassium hydroxide that can be readily removed by rinsing with water. Similar to the surfactant‐free aqueous dispersions previously developed for graphene and SWCNTs, the nanothreads remain stably dispersed, free of visible aggregation, and optically active (Figures S7 and S8, Supporting Information), indicating that their surface charge and chemical functionality provide effective colloidal stabilization. The use of water as a dispersion medium not only simplifies solvent removal but also enables the fabrication of uniform thin films by scalable techniques such as vacuum filtration and stamping, thereby allowing the fabrication of thin films and investigation of their solid‐state properties as described in the following sections.

### 2.5 Assembly and Characterization of Carbon Nitride Nanothread Thin Films

**FIGURE 4:**
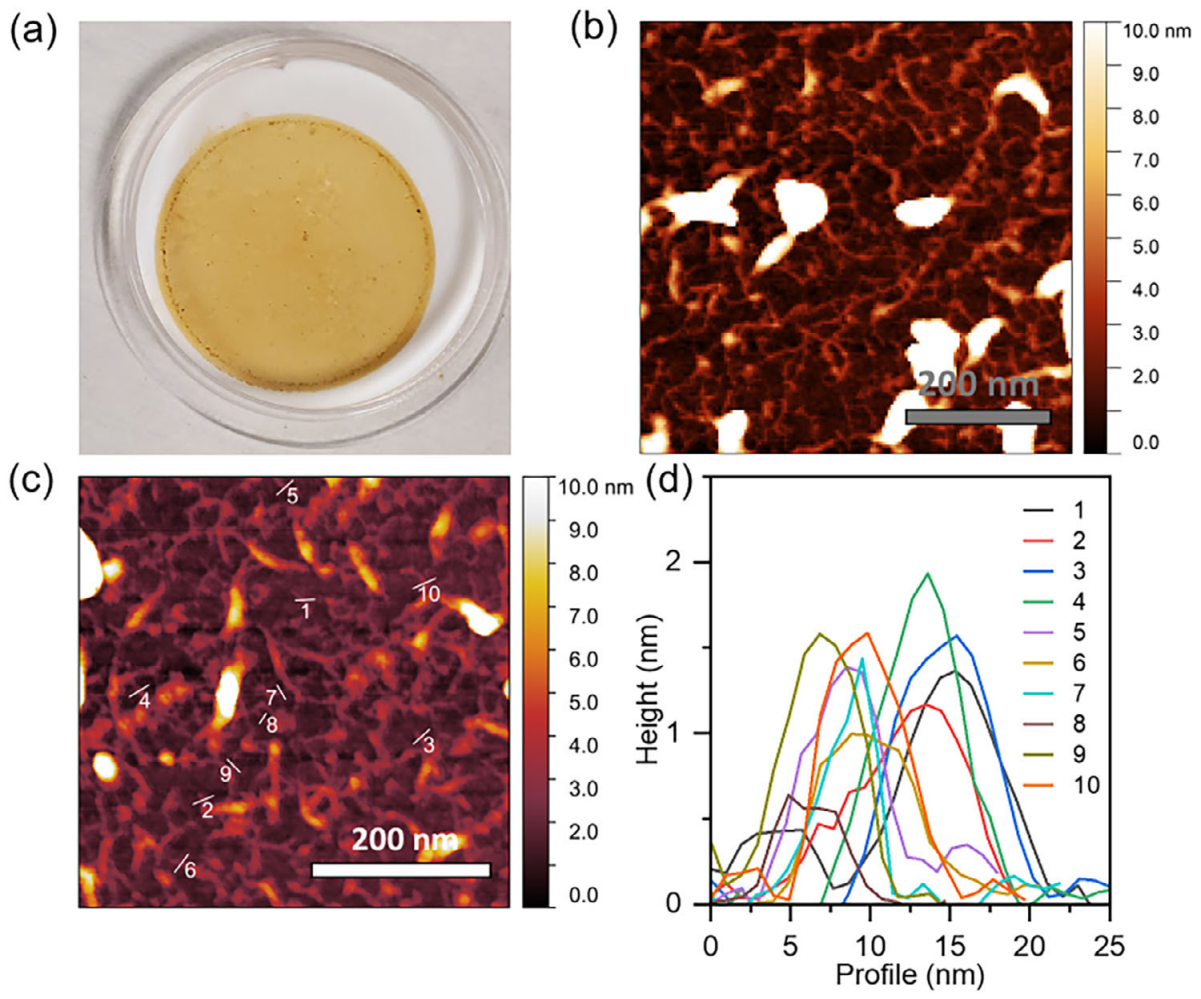
Morphology of deposited carbon nitride nanothread thin films. (a). Photograph of a carbon nitride nanothread thin film collected on a filtration membrane. The film can be readily transferred onto various substrates through stamping for characterization and device fabrication. (b) Atomic‐force microscopy (AFM) micrograph of the carbon nitride nanothread film deposited on a Si substrate, showing a network of long, thin objects made up of individual and small bundles of carbon nanothreads. (c) Enlarged AFM micrograph of carbon nitride nanothreads. (d) AFM height profiles taken directly from the bare Si substrate to the top of each feature labeled 1–10 in (c). These height traces confirm that the measured values correspond to isolated nanothreads resting on the substrate surface rather than to surface roughness of a continuous film. Most features exhibit heights below 2 nm, consistent with individualized nanothreads and very small bundles, while occasional bundles reach heights up to ∼4 nm. A more complete height distribution is provided in Figure S10 (Supporting Information).

Following their dispersion in various solvents, exfoliated nanothreads can be deposited onto substrates for further characterization. Thin films of individualized carbon nitride nanothreads were produced by vacuum filtration of their aqueous dispersion through a nitrocellulose filter membrane with 25 nm nanometer‐sized pores (Millipore). The trapped carbon nanothreads films were thoroughly rinsed with deionized water to remove residual potassium hydroxide, yielding a clean film composed sorely of carbon nitride nanothreads. Figure 4a shows a nanothread thin film covering 10 cm^2^ of the filter. This film was transferred onto various substrates by stamping the membrane filter with the film side down and dissolving the membrane with acetone, leaving a thin film of dispersed material. To verify whether potassium ions remain in the exfoliated product, XPS analysis was performed on the carbon nitride nanothread films after washing and transfer (Figure S9, Supporting Information). The absence of any detectable K 2p signal confirms that K^+^ ions are completely removed during the purification steps, indicating that potassium functions solely as a transient charge‐transfer mediator during intercalation/exfoliation and does not remain in the final nanothread film.

TEM imaging of the individualized nanothreads was attempted; however, as discussed previously and shown in Figure S3 (Supporting Information), the as‐synthesized nanothreads are highly beam sensitive and readily lose their structural integrity even under low electron‐dose conditions. The isolated nanothreads obtained after exfoliation, which are thinner and less constrained, are expected to be even more susceptible to beam‐induced damage. As a result, it was not possible to obtain meaningful TEM images of the exfoliated material. For individualized nanothreads, AFM therefore provides a more reliable and non‐destructive characterization method, since it allows accurate measurement of height, length, and morphology without damaging the structures.

An AFM image of the transferred film on a Si substrate (Figure 4b,c) reveals a network of long, thin, high‐aspect‐ratio objects, each presumably an individual nanothread or a small bundle of nanothreads. AFM height profiles were measured directly from the substrate surface to the top of each isolated feature, enabling unambiguous determination of individual thread and bundle thicknesses. The thinnest structures exhibit heights of ∼0.6 nm (Figure 4c,d), which is consistent with the expected diameter of a single carbon nitride nanothread [5]. Because these measurements reference the bare substrate rather than the film surface, they confirm that the observed features correspond to individualized and small bundles of nanothreads rather than roughness or aggregated domains. The length of the 1D nanothread features ranges from ∼50 to several hundred nanometers (Figure S10a, Supporting Information), and the height distribution indicates that the majority of exfoliated nanothreads are individualized or present as small bundles consisting of 2−5 threads (Figure S10b, Supporting Information).

**FIGURE 5:**
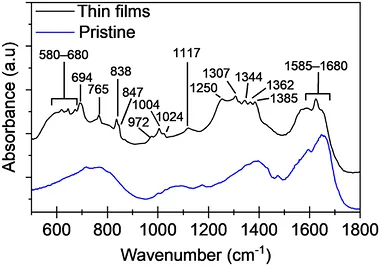
Spectroscopy of carbon nitride nanothread films. FTIR spectrum of film deposited from the suspension of individualized carbon nitride nanothreads compared with the spectrum of the pristine material. The FTIR spectra of carbon nitride nanothread film after dispersion and processing exhibit sharper and more defined IR features. This suggests improved structural order in the processed film.

Figure 5 compares the FTIR spectra of the as‐synthesized pristine sample and the processed, transferred thin‐film sample. In contrast to the pristine sample, the thin film exhibits sharper and better‐defined IR features, indicating increased chemical uniformity after processing. For example, the radial breathing modes at 580–680 cm^−1^ and the axial inter‐ring elongation‐compression mode, corresponding to inter‐ring C─N stretching between C_5_H_5_N units, around 1117 cm^−1^ are more prominent in the thin‐film sample. The sp^3^ C─H stretching mode (2850–2960 cm^−1^) and C═C/C═N stretching modes (1585–1680 cm^−1^) are also visible in the thin‐film sample (Figure S2, Supporting Information), indicating that both saturated and partially saturated segments of carbon nitride nanothreads remain after processing. IR peaks at 717, 771, and 1473 cm^−1^, commonly associated with amorphous carbon nitride, are significantly weakened or absent in the thin‐film sample [5, 37, 38]. The O−H/N−H stretching features around 3000 cm^−1^, are also greatly diminished in the thin film sample (Figure S2, Supporting Information). These changes indicate that intercalation, exfoliation, and transfer selectively enrich the nanothread fraction while removing amorphous carbon nitride content.

**FIGURE 6:**
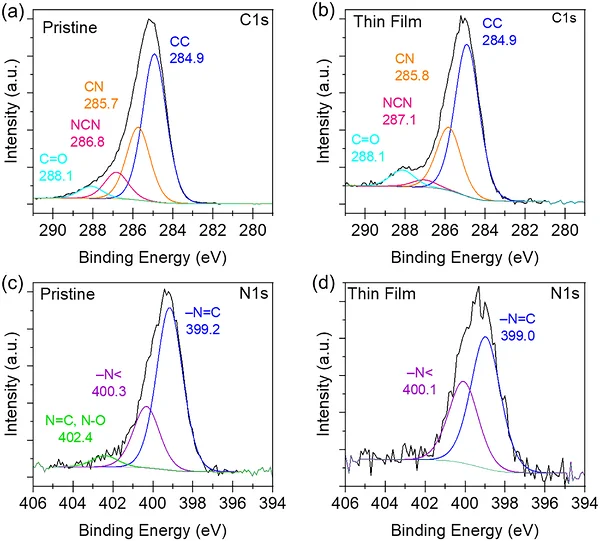
X‐ray photoelectron spectroscopy (XPS) of carbon nitride nanothread samples before and after processing. C1s spectra of (a) the as‐synthesized sample and (b) the thin film sample obtained from the nanothread dispersion. Also, N1s spectra of (c) the as‐synthesized sample and (d) the thin film sample. The raw data is plotted in black lines and fit curves are in different colors. The high‐resolution C1s and N1s spectra of both the pristine sample and the film exhibit similar peak shapes and positions. The XPS data provides evidence that the dispersion and individualization process does not lead to undesirable chemical modifications of the carbon nitride nanothreads, but aids in the purification of the samples.

XPS was used to study the chemical state of the nanothread samples before and after processing. The high‐resolution C1s spectra of the as‐grown and thin‐film samples exhibit similar shape and peak positions (Figure 6a,b), with peaks assigned to carbon species with no bonded nitrogen atom (284.9 eV), one bonded nitrogen atom (285.8 eV) and two bonded nitrogen atoms (287.1 eV) [5]. The high‐resolution C1s spectra of the as‐grown and thin‐film samples exhibit similar shape and peak positions (Figure 6a,b), with peaks assigned to carbon species with no bonded nitrogen atom (284.9 eV), one bonded nitrogen atom (285.8 eV) and two bonded nitrogen atoms (287.1 eV). As sp^2^ and sp^3^ carbon often differ only slightly in binding energy (c.a. 0.7 eV), it is difficult to distinguish the sp^2^ and sp^3^ contributions to these peaks. The peak at 288.1 eV corresponds to carboxylic acid groups caused by surface oxidation. The peak assigned to carbon atoms bonded with two nitrogen atoms (287.1 eV) is much weaker in the thin‐film sample, which suggests the selective removal of certain species such as amorphous byproducts or highly defective threads during processing.

The high‐resolution N1s spectrum (Figure 6c,d) of the as‐grown sample shows peaks at 399.2, 400.3, and 402.4 eV, while the thin‐film sample shows peaks only at 399.0 and 400.1 eV. Based on peak assignments for common nitrogen‐containing species, the peak near 399.0 eV is assigned to two‐coordinate nitrogen (as in pyridinic nitrogen or imine), while the peak near 400.1 eV is assigned to three‐coordinate nitrogen (as in amine or pyrrolic nitrogen) [63, 64, 65, 66]. The peak at 402.4 eV is attributed to N–O species and N═C species with a delocalized nitrogen lone pair, likely related to byproducts or defect sites in nanothreads or oligomers [67]. The relative intensity of the two‐coordinate nitrogen species, compared to the three‐coordinate ones, is lower in the thin‐film sample. This observation may suggest the selective removal of components that have high degree of unsaturation, which is expected for amorphous byproducts, oligomers, and some partially saturated nanothreads.

Overall, XPS and FTIR evidence suggest that the dispersion and individualization process does not significantly alter the chemical structure of the nanothread sample, but improves structural order, likely through the selective removal of less well‐ordered and/or incompletely polymerized species from the as‐grown sample. Species with a larger fraction of unreacted sp^2^ carbon or nitrogen, such as amorphous byproducts or oligomers, should dissolve more readily than more rigid and more fully saturated nanothread species. As such, ultracentrifugation helps separate individualized and dispersed nanothreads from other components. Collectively, AFM, FTIR, and XPS provide complementary structural and spectroscopic evidence supporting the conclusion that the processed material consists of individualized nanothreads and small bundles. These results demonstrate the first post‐synthetic route to purify and individualize nanothread samples, a key step toward the utilization of nanothread materials and detailed study of their structure/property relations.

### 2.6 Transport Properties of Carbon Nitride Nanothread Thin Films

**FIGURE 7:**
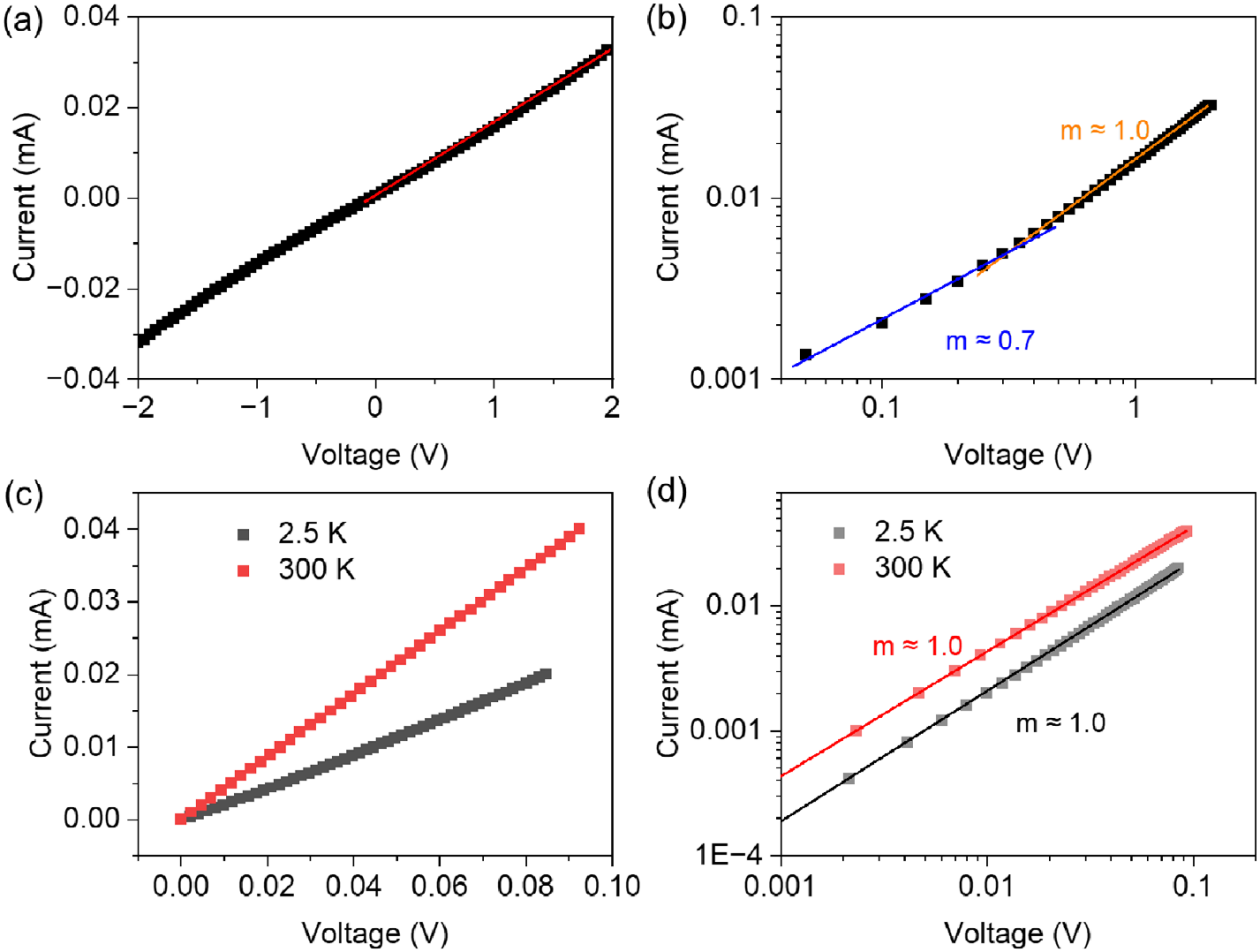
Electrical transport in solution‐processed carbon nitride nanothread thin films. (a) *I–V* characteristics of the as‐deposited nanothread film measured using interdigitated Au electrodes (3 µm gap). (b) Log–log plot of the *I–V* data showing sublinear transport at low bias (𝑚 ≈ 0.7) and nearly ohmic conduction at higher bias (𝑚 ≈ 1.0). (c) Low‐bias *I–V* characteristics of the thermally annealed film measured at 2.5 and 300 K. (d) Corresponding log–log plots showing (𝑚 ≈ 1.0) at both temperatures, consistent with ohmic transport after annealing and indicating that the low‐bias nonlinearity in the as‐deposited films originates primarily from extrinsic processing‐related effects.

Transport properties of carbon nitride nanothread thin films were investigated using devices fabricated by depositing the nanothread films onto interdigitated gold electrodes with a 3µm gap. The short distance between the electrodes improves the likelihood of obtaining a percolating network of nanothreads in these thin films (<6 nm thick). The measured *I–V* characteristics of an as‐deposited nanothread film exhibit a symmetric and nearly linear response over the applied bias range (Figure 7a), indicating efficient charge injection and the formation of nearly ohmic contacts between the carbon nitride nanothread film and the Au electrodes.

Further analysis using a log‐log representation (Figure 7b) reveals a power‐law dependence of the current on voltage, 𝐼 ∝ 𝑉^𝑚^, where 𝑚 ≈ 0.7 in the low bias regime (< 0.25 V). The sublinear behavior observed at low bias suggests non‐ideal carrier transport arising from localized barriers within the nanothread film. Such behavior is likely associated with residual solvent molecules and amorphous carbonaceous species trapped at nanothread‐nanothread and nanothread film‐electrode interfaces during solution processing. These structural and interfacial inhomogeneities can introduce localized trap states and tunneling barriers that suppress charge transport under weak electric fields. At higher bias (> 0.25 V), these barriers are progressively overcome, resulting in a transition to nearly ohmic conduction (m ≈ 1).

To improve electrical transport, the films were subjected to mild thermal annealing at 200°C in a low‐pressure CVD furnace under a flowing Ar/H_2_ atmosphere. Thermal annealing is expected to remove residual solvent and amorphous carbonaceous species while enhancing electrical coupling at nanothread‐nanothread and nanothread‐electrode interfaces. Indeed, after annealing, resistance at room temperature decreased by approximately an order of magnitude. The *I–V* characteristics measured at low bias after thermal treatment exhibit a linear response (Figure 7c) at 2.5 and 300 K, indicating efficient charge injection and improved charge transport through the nanothread network. Log–log representation (Figure 7d) reveals a power‐law dependence of the current on voltage, 𝐼 ∝ 𝑉^𝑚^, with m ≈ 1.0 at both temperatures. This linear behavior confirms ohmic conduction at low bias following thermal annealing.

**FIGURE 8:**
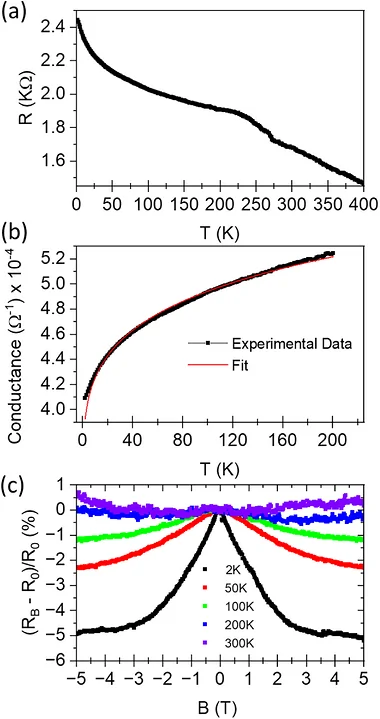
Temperature‐dependent transport and magnetoresistance of carbon nitride nanothread thin films. (a) Temperature‐dependent resistance measurements (*R–T*) showing a weakly semiconducting behavior. (b) Plot of conductance as a function of temperature T from 2 to 200K, fitted using the weak localisation model. The good fit of the results from 2 to 200 K suggest quantum effects begin to dominate the conduction mechanism in the carbon nanothread films at these relatively high temperatures. (c) Magnetotransport (MR) measurements of nanothread thin film under magnetic field ranging from −5 to 5T and at temperature ranging from 2 to 300 K. The carbon nitride nanothread thin film has a negative magneto resistance at 2K, which gradually decreases as the sample warms up from 2K to 100K. At 300K, a clearly positive magneto resistance is observed.

The resistance was measured from 2 to 400 K using a two‐probe technique (Figure 8a) [56]. Remarkably, the resistance displays only a weak upturn upon cooling to 2 K, with a residual resistivity ratio that compares favorably to those of highly conductive polymers. The qualitative conclusion of a very low residual resistance ratio in the nanothread film is robust against the two‐probe nature of the measurement, because any strong upturn in film resistance upon cooling would be visible in series with the contact resistance. Temperature‐dependent transport measurements reveal a weakly semiconducting behavior in the nanothread film, with the resistance rising smoothly by only ∼40% from 300 to 2 K (Figure 8a). The shallow temperature dependence indicates that the nanothread film contains a considerable amount of sp^2^‐hybridized components, such as degree 4 or degree 2 nanothread segments, and that charge transport proceeds between these sites through minimal hopping barriers; this is consistent with the efficacy of alkali intercalation in thread dispersal. The apparent discrepancy between the large optical bandgap (2.57–3.37 eV) and the weakly semiconducting electrical response likely arises from the different ways in which the optical and transport gaps are measured. The optical bandgap, determined from absorption spectroscopy, reflects allowed electronic transitions within the carbon nitride nanothread framework, whereas the transport gap, derived from the electrical measurement, is governed by carrier mobility and strongly influenced by structural disorder and defect‐induced localized states. In particular, sp^2^ sites within the nanothreads, which are abundant in our samples as evidenced by FTIR and XPS analyses, are expected to introduce states that facilitate charge transport despite a wide optical gap [68].

Above ∼225 K, the resistance shows stronger noise‐like variations around the main trend with a weak hysteresis, see Figure S11 (Supporting Information). These characteristics may reflect temperature‐dependent variations in bundle‐bundle or bundle‐electrode contact due to sliding caused by differential thermal expansion. For this reason, we do not perform a quantitative analysis of the temperature dependence in this temperature range, noting only that it is relatively weak and broadly consistent with conduction processes familiar from conductive polymers or nanotube networks, [69, 70, 71, 72, 73, 74, 75] such as 2D variable‐range hopping (VRH) (also seen in disordered thin films) [76, 77, 78], a reduced activation energy [79] or fluctuation‐induced tunneling [80].

The resistance variation below ∼225 K is smooth and shows a very small residual resistance ratio. We analyze conductance (G) as a function of temperature from 2 to 200 K (Figure 8b) using a weak localization (WL) model [81, 82, 83] of the form G = G_o_ + *aT* ^*p*/2^ + *bT* ^1/2^ where Go = 3.91 × 10^−4^ (1/Ω), *a* = 3.39 × 10^−5^ (C^4^·s^2^/kg^2^·m^6^), *b* = 5.60 × 10^−6^ (C^4^·s^2^/kg^2^·m^6^·K), and *p* = 0.34, the first term represents Drude conductance while the second and third terms account for weak localization and electron‐electron interaction, respectively. The model demonstrated an excellent fit across the temperature range (2–200 K), suggesting that quantum effects play a dominant role in conduction with significant contribution from electron‐electron interactions at low temperatures, which has also been reported in highly conducting conjugated polymers, which share structural similarities with nanothreads [84, 85, 86, 87]. Using the value of *a* obtained from the fitting of the G‐T data using the WL model, the phase coherence length (*L*_ø_) was calculated to be 54.5 nm, using the equation, $a=e^{2}s/2l\pi^{2}\hbar L_{\emptyset }$, where *s* is the cross sectional area and *l* is the length of the sample. The phase coherence length is ≈3 times smaller than the nano thread lengths > 200 nm which means weak localization is a plausible transport mechanism in the nanothread films.

Magneto transport (MR) measurements were conducted under a magnetic field ranging from −5 to 5T and at temperatures ranging from 2 to 300 K (Figure 8c). At 2K, the nanothread films have a negative magnetoresistance which diminishes in magnitude as the temperature increases, transitioning to a small positive magnetoresistance around 200K. Weak localization effects in conductive polymers (including highly conductive polyacetylene [88, 89, 90, 91] and carbonized ion‐implanted polymers^70^) can produce a negative magnetoresistance [83], and similar behavior is also seen due to weak localization in networks of SWCNTs [70]. Negative magnetoresistance may also be obtained from orbital contributions in a variable‐range hopping regime [92], but the sign of these contributions can reverse at higher conductivities, opposite to the observed trend. At 300 K, the positive magnetoresistance is well fit by a classical quadratic model. Note that gold (the electrode material) has a small positive magnetoresistance.

## 3 Conclusion

Carbon nanothreads are an emerging class of 1D nanomaterials that promise exceptional mechanical strength and tunable physical properties via precursor design. The bundled nature of carbon nanothreads in high‐pressure synthesized samples has posed challenges in their handling, processing, and characterization, thus hindering the full exploration of their potential. In this study, we demonstrated the individualization of carbon nitride nanothreads through reductive dissolution. This represents the first successful individualization and dispersion of nanothread materials, with the methodology potentially applicable to other nanothread types. The resulting dispersions of carbon nitride nanothreads absorb in the 300–450 nm range and show blue/green photoluminescence. Percolating thin‐film networks of small bundles and individual nanothreads exhibit near‐metallic conduction. The ability to solution‐process and deposit purified and individualized nanothreads onto various substrates expands opportunities for advanced property characterization and broadens the scope for their application.

Beyond carbon nitride nanothreads, the reductive dissolution strategy demonstrated here may provide a general route toward the individualization and processing of other nanothread materials, including those derived from benzene, furan, and polycyclic aromatic hydrocarbons. Notably, we have shown in this work that the approach is also applicable to furan‐derived nanothreads, suggesting that reductive dissolution is not limited to a single nanothread chemistry. The applicability of this approach to other nanothread systems will depend on the ability of the nanothread framework to accept and stabilize injected charge without undergoing structural degradation. Alternative strategies, including electrochemical intercalation, covalent or non‐covalent functionalization, and chemical modification of residual unsaturated carbon sites, may further enhance nanothread solubility and processability. In particular, electrochemical intercalation offers a potentially milder route to expand inter‐thread spacing and weaken inter‐thread interactions while minimizing structural damage, analogous to recent advances in the isolation of single‐chain van der Waals materials [93]. These complementary approaches may ultimately enable the dispersion, purification, and integration of a broader range of nanothread materials into functional composites and devices.

By overcoming the challenges associated with bundling and processing, this study paves the way for harnessing the exceptional properties of carbon nanothreads in diverse fields, which may include electronics, optoelectronics, sensors, nanocomposites, and energy harvesting devices.

## 4 Experimental Section

### 4.1 Synthesis of Carbon Nitride Nanothreads

Carbon nitride nanothreads were synthesized by compressing ca. 20 mg of pyridine in a PE press up to 23 GPa at room temperature as reported by Li et al. [5]. The precursor was purchased from Sigma‐Aldrich (Pyridine anhydrous, 99.8%) and used without additional purification. The compression rate was 4–5 GPa/h below 10 GPa, 3–4 GPa/h below 14 GPa, 2–3 below 19 GPa and 0.6‐1.2 GPa/h up to the maximum pressure. After holding for 1–2 h at the maximum pressure, the sample was decompressed at similar rates. A dark orange solid product was recovered after decompressing to ambient conditions. Each synthesis run typically yields about 2–6 mg of solid product.

### 4.2 X‐Ray Diffraction

X‐ray diffraction of the recovered carbon nitride nanothread product was measured by in‐house X‐ray diffractometer equipped with Rigaku MicroMax‐007HF X‐ray generator and Rigaku HyPix‐Arc 150° detector. A Cu rotation anode (λ = 1.54 Å) was used as the X‐ray source.

### 4.3 FTIR Spectroscopy

FTIR spectrum of the as‐synthesized sample was collected on a Bruker Vertex V70 spectrometer equipped with a Hyperion 3000 FT‐IR microscope. FTIR spectrum for carbon nitride nanothread thin film on Si substrate was collected using ATR‐FTIR with diamond as the ATR crystal.

### 4.4 Transmission Electron Microscopy

TEM was performed using a FEI Titan G2 60–300 microscope, with spherical aberration correction. Samples were imaged on a copper grid with lacey carbon.

### 4.5 Thermal Gravimetric Analysis

Thermal gravimetric analysis/mass spectrometry (TGA‐MS) was performed on Discovery TGA 5500 coupled with Discovery MS (TA Instrument). The sample was first purged with Argon for an hour and then measured at a temperature ramp of 10°C/min up to 700°C.

### 4.6 Reductive Dissolution of Carbon Nitride Nanothreads

Potassium salt of carbon nitride nanothreads preparation and dissolution experiments were carried out inside an argon‐filled glovebox environment. In a typical reaction, ∼1 mg of freshly cut potassium metal (K) was added to a glass vial containing about 9 mg carbon nitride nanothread sample. The mixture of K and nanothread sample was heated on a hot plate at 150°C while continuously mixed for 10 min using a stainless‐steel spatula. The vial was left on the hot plate, and the mixture was mixed with the stainless‐steel spatula at every 10 min interval for 1 h and then every 30 min for 4 h. Afterward, the product (the potassium salt of nanothreads) was allowed to cool to room temperature, and the vial was tightly capped for later use. The reduction process was also performed on a furan derived carbon nanothread sample, for XRD characterization. To prepare nanothread dispersion, 3 mg of potassium salt of carbon nitride nanothreads was added to 15 mL of dry analytical‐grade NMP, THF, or DMSO solvent (or 0.2 mg/mL) inside the glovebox. The potassium salt of carbon nitride nanothreads dissolved immediately and formed an orange dispersion. The dispersion was tightly capped and mixed on a stir plate (at 400 rpm) for 24 h with a Teflon magnetic stirrer. After stirring, the dispersion was left still overnight to allow large undissolved particles to settle at the bottom. After removing the large particles following a mild centrifugation at 1000 rpm, the dispersion was centrifuged in 3 mL vials at 12 000 rpm for 1 h, and then the precipitate was collected and redispersed in a desired solvent for characterization and thin film fabrication.

### 4.7 Absorption Spectroscopy

Absorption spectra of a dispersion of nanothreads in NMP were recorded in 10 mm optical path quartz cuvettes using a LAMBDA 950 UV–vis–NIR spectrometer (JascoV‐670) in the wavelength range of 350–800 nm. A baseline correction was performed using DMSO.

### 4.8 Photoluminescence Excitation Experiments

Photoluminescence (PL) spectra were acquired by using a fluorescence spectrometer (Edinburgh Instrument FLS 1000) at room temperature with a 280, 300, 320, 350, and 370 nm excitation.

### 4.9 Thin Films

Carbon nitride nanothread thin films were prepared on different substrates by vacuum filtration and stamping [94]. Using vacuum filtration, 5 mL of a suspension of carbon nanothreads in DMSO was diluted into 50 mL of distilled water and filtered through a 25 nm pore size nitrocellulose membrane filter (Millipore). A further 250 mL of distilled water was filtered through the nitrocellulose membrane to wash off some remaining KOH and NMP from the carbon nitride nanothreads. The carbon nitride nanothread films were deposited on carefully cleaned Si and interdigitated electrons on Si/SiO_2_ substrates by stamping. The nitrocellulose membrane was then dissolved by placing the films successively in several acetone, methanol, and water baths for 15 minutes in each bath. The thin films were then annealed at 200°C in a CVD furnace under an Ar/H_2_ flow. The thin films on Si were used for AFM, FTIR, and XPS characterization.

### 4.10 AFM Measurements

Topographical imaging of carbon nitride nanothread films transferred onto Si substrates by stamping was performed using a Bruker Dimension Icon atomic force microscope operated in PeakForce Tapping mode.

### 4.11 XPS Measurements

XPS experiments were performed using a Physical Electronics VersaProbe III instrument equipped with a monochromatic Al k_α_ x‐ray source (hν = 1,486.6 eV) and a concentric hemispherical analyzer. Charge neutralization was performed using both low energy electrons (< 5 eV) and argon ions. The binding energy axis was calibrated using sputter‐cleaned Cu (Cu 2p_3/2_ = 932.62 eV, Cu 3p_3/2_ = 75.1 eV) and Au foils (Au 4f_7/2_ = 83.96 eV). Peaks were charge referenced to CH_x_ band in the carbon 1s spectra at 284.8 eV.

### 4.12 Transport Measurements

Transport measurements were made on the carbon nitride nanothreads deposited on the interdigitated electrodes. Current‐voltage measurements (Figure 7a) were performed on as‐deposited and room temperature vacuum dried carbon nanothread film, using a Keithley 2400 SourceMeter. The bias was swept from −2 to +2 V and subsequently from +2 to −2 V using a voltage step size of 0.05 V. Forward and reverse voltage sweeps produced overlapping *I–V* characteristics, indicating negligible hysteresis. Low‐bias *I–V* measurements (Figure 7c) were performed in current‐source mode by sourcing current from 0.4 to 20 µA in increments of 0.4 µA while recording the corresponding voltage response. The measured current and voltage values were then used to construct the low‐bias *I–V* curves. The use of current‐source mode for the low‐bias measurements was adopted to improve measurement sensitivity in the low‐current regime. Temperature dependent transport measurements were made on the carbon nitride nanothreads deposited on the interdigitated electrodes. The transport measurements were performed in a standard two‐terminal configuration from 2 to 400 K with a Quantum Design Dynacool Physical Properties Measurement System (PPMS). The resistance was measured using the built‐in DC resistivity module of the PPMS with an alternating DC drive current of 1µA. The magnetic field was applied perpendicular to the device plane. The PPMS instrument DOI is 10.60551/rxfx‐9h58.

### 4.13 Density Functional Theory Calculations

DFT calculations employed the VASP [52, 53, 54, 55, 56] package, using the Perdew–Burke–Ernzerhof exchange correlation functional [49], and Projector Augmented Waves [52, 95]. The simulations for thread packing were performed with a plane wave energy cutoff of 600 eV and a 11×11×3 Γ‐centered k‐point mesh. The electronic energy and interatomic forces were converged to 10^−6^ eV and 0.01 eV/Å. For the nonlocal vdW‐DF method the optB86b exchange functional was used [96]. The intercalation simulations were performed with a plane wave energy cutoff of 550 eV and a Γ‐centered k‐point mesh with step size greater than 2π/24 Å^−1^ The electronic energy and interatomic forces were converged to 10^−5^ eV and 0.01 eV/Å. DFT‐B3 with Becke‐Johnson damping was used to model dispersion forces [50, 51].

### 4.14 Supporting Information

XRD, FTIR, TEM TGA characterization of as synthesized carbon nitride nanothreads; XRD analysis of alkali intercalated furan‐derived carbon nanothreads; Computational details of the intercalation mechanism, UV–vis absorption data of furan‐derived carbon nanothreads dispersed in DMSO; photographs of suspensions of carbon nitride nanothreads in different solvents, UV‐vis absorption data of carbon nitride nanothreads compared to that of furan‐derived carbon nanothreads dispersed in DMSO; XPS spectrum of the K 2p and C 1s regions of the carbon nitride nanothread films; AFM analysis of length and height of carbon nanothreads; and electrical characterization data of the carbon nitride nanothread films.

## Author contributions

G.B., V.H.C., and M.T. conceived the idea. S.W. and G.B. synthesized all the samples, characterized all the materials, and analyzed the results. B.X. and V.H.C. performed the first‐principles calculations and provided theoretical insight. Y.L. assisted with material characterization and performed the TGA and AFM measurements. M.M. and Z.Y. assisted with AFM measurements and analysis. Á.E.E.C fabricated the devices for transport studies using vacuum filtration and stamping. D.Z. performed transport measurements, and G.B. and G.C. analyzed the transport data. G.B., S.W., B.X., and G.C. wrote the draft manuscript, and V.H.C. and M.T. revised it. All co‐authors commented on the manuscript before its submission.

## Conflicts of Interest

The Pennsylvania State University has filed a patent application (US Patent Application, 63/267,561 (2022)) that lists G.B., S.W., Y.L., N.P.L, V.C., and M.T. as inventors. Other than this, the authors declare no competing interests.

## Data and Materials Availability

All data needed to evaluate the conclusions in the paper are present in the paper and/or the Supplementary Materials. Additional data related to this paper may be requested from the authors.

## Supporting information




**Supporting File**: smll74445‐sup‐0001‐SuppMat.pdf.

## Data Availability

The data that support the findings of this study are available from the corresponding author upon reasonable request.
