# Supplementary material for: Individualization and Purification of Carbon Nanothreads
Source: Small. 2026 Aug 3;22(48):e74445. doi: 10.1002/smll.74445 (PMC13509114; doi:10.1002/smll.74445)
Supplement: Supplementary file 1 [file SMLL-22-e74445-s001.pdf]

Individualization and Purification of Carbon Nanothreads

George Bepete[1,2,3,4,5,6*], Sikai Wu[2], Bohan Xu[1], Ámbar E. Escobar Colón[1], Andres Fest[7], Da Zhou[1], Yu Lei[1,4,8], Methembe Moyo[6], Zhuohang Yu[3,4,7], George Chimowa[9], Kazunori Fujisawa[10], Nestor Perea-Lopez[1,4], Vincent H. Crespi[1,2,4,7**] and Mauricio Terrones[1,2,3,4,7, ***]

[1]Department of Physics, The Pennsylvania State University; University Park, Pennsylvania 16802, USA.

[2]Department of Chemistry, The Pennsylvania State University; University Park, Pennsylvania 16802, USA.

[3]Center for 2-Dimensional and Layered Materials, and Centre for Atomically Thin Multifunctional Coatings, The Pennsylvania State University, University Park, Pennsylvania 16802, USA.

[4]Materials Research Institute, The Pennsylvania State University, University Park, Pennsylvania 16082, USA.

[5]Department of Chemical and Materials Engineering, and Centre for NanoScience Research (CeNSR), Concordia University, 7141 Sherbrooke Street West., Montreal H4B 1R6, Canada.

[6]Department of Physics, and Centre for NanoScience Research (CeNSR), Concordia University, 7141 Sherbrooke Street West., Montreal H4B 1R6, Canada.

[7]Department of Materials Science and Engineering, The Pennsylvania State University; University Park, Pennsylvania 16802, USA.

[8]Institute of Materials Research & Shenzhen Key Laboratory of Advanced Layered Materials for Value-added Applications & Center of Double Helix & Guangdong Provincial Key Laboratory of Thermal Management Engineering and Materials, Tsinghua Shenzhen International Graduate School, Shenzhen, Guangdong 518055 China.

[9]Department of Physics and Astronomy, Botswana International University of Science and Technology, P. Bag 16, Palapye 0267, Botswana.

[10]Research Initiative for Supra-Materials and Global Aqua Innovation Centre, Shinshu University; 4-17-1Wakasato, Nagano 380-8553, Japan.

Additional Discussion

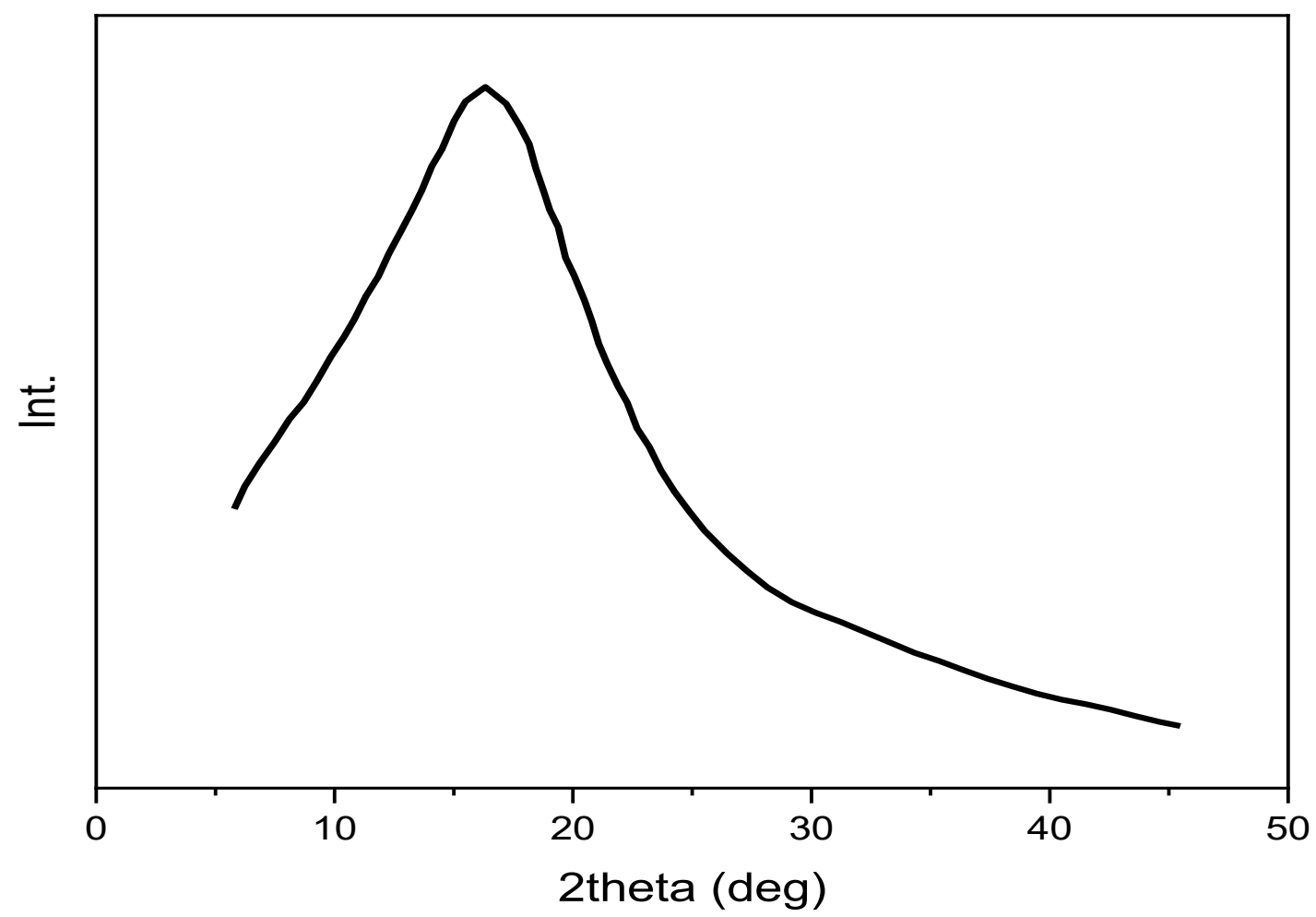

Figure S1. 1-Dimensional diffraction pattern of carbon nitride nanothreads recovered from the compression of pyridine in a Paris-Edinburgh press. (1-D line profile extracted from Figure1c.)

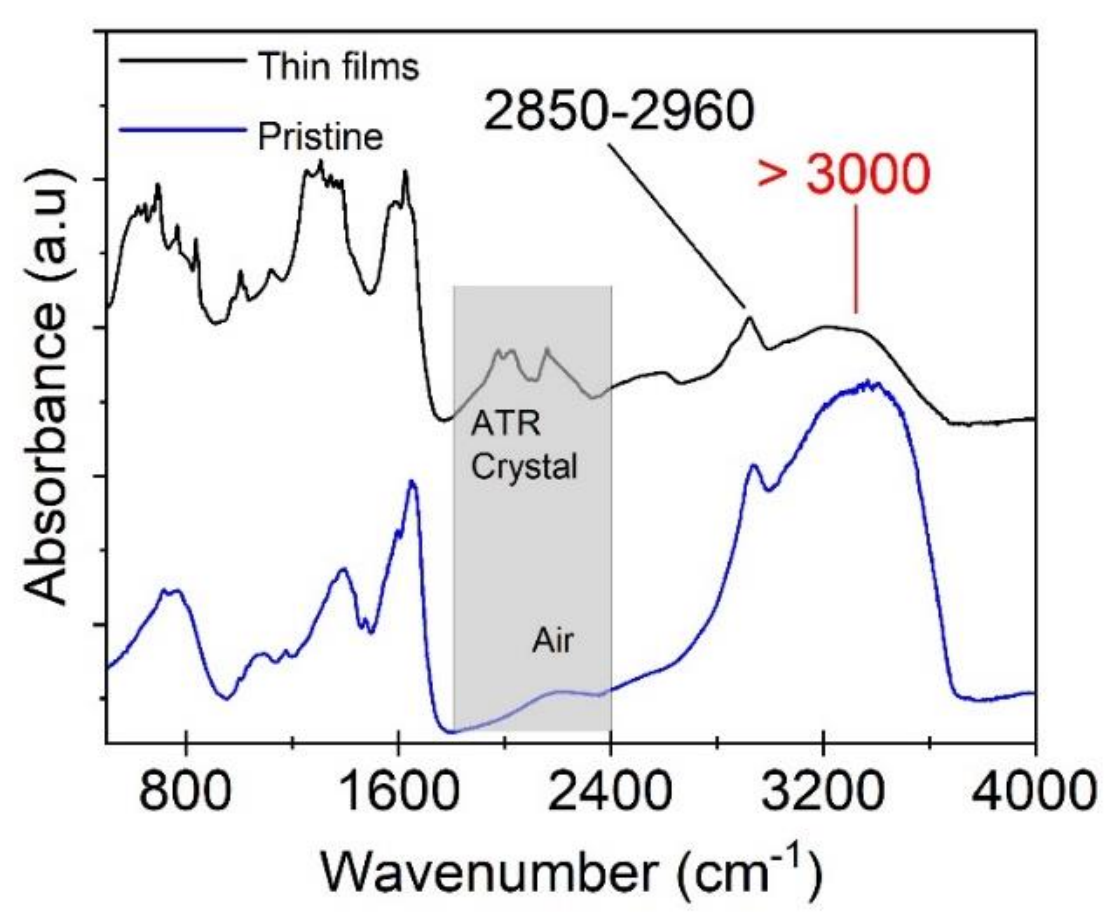

Figure S2. FTIR spectrum of thin film deposited from the suspension of individualized carbon nitride nanothreads compared with the spectrum of the pristine material. The FTIR spectra of carbon nitride nanothread film after dispersion and processing exhibit sharper and more defined IR features. This suggests improved structural order and the absence of amorphous carbon materials in the processed film.

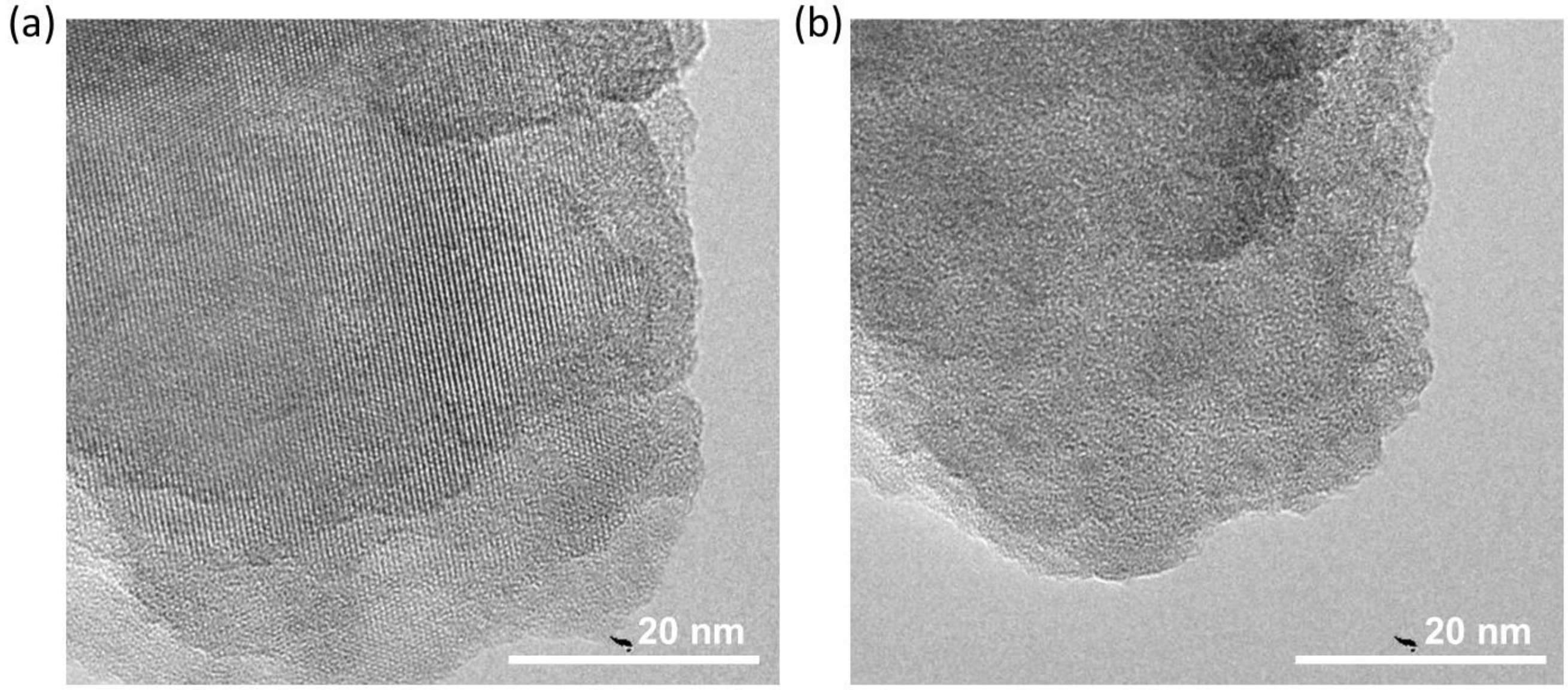

Figure S3. TEM images illustrating the beam sensitivity of the carbon nitride nanothread sample. (a) Ordered nanothread region showing well-defined lattice fringes. (b) The same region after brief exposure to the electron beam, showing the complete loss of fringe contrast and the development of a featureless, amorphous appearance due to rapid beam-induced damage.

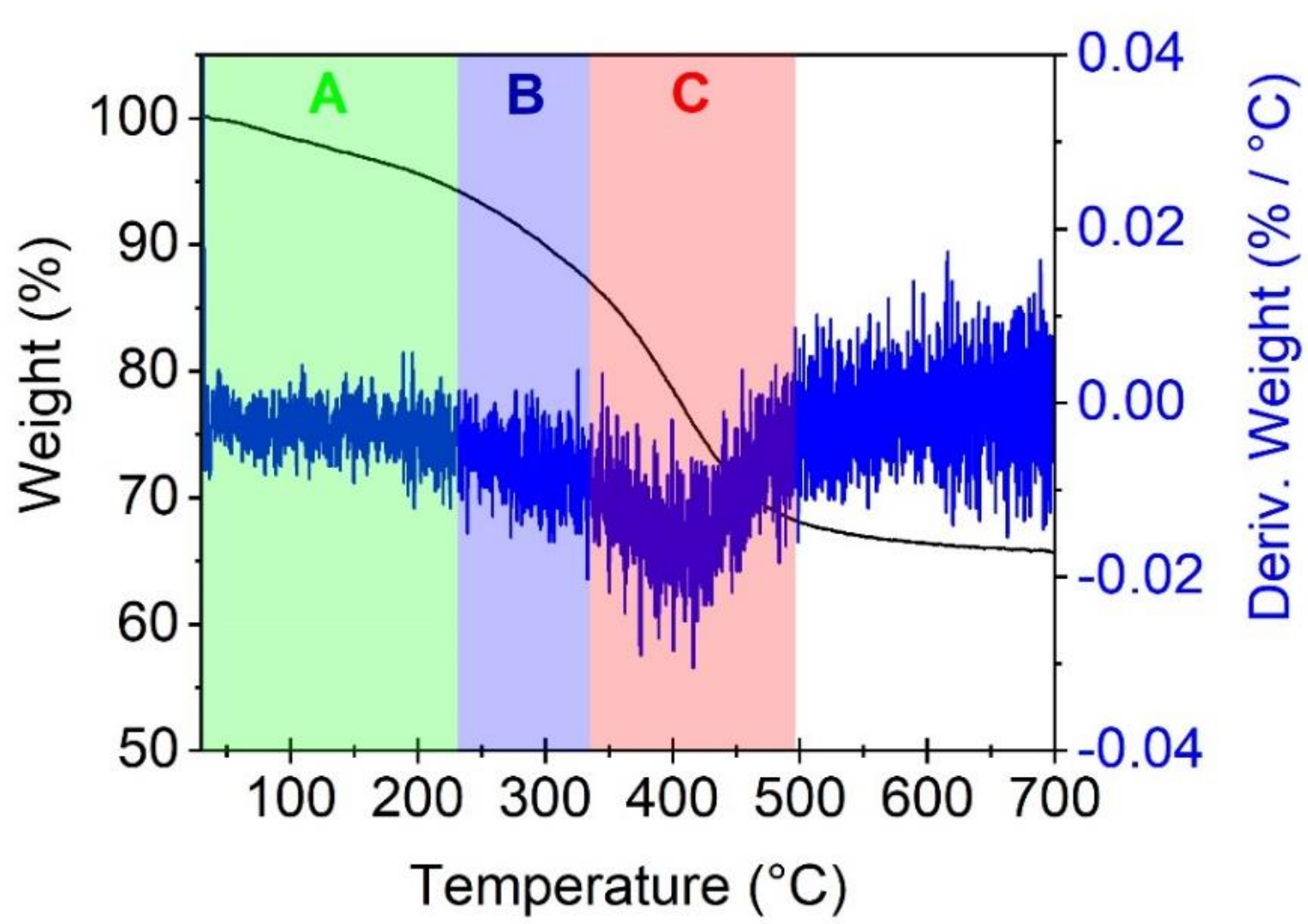

Figure S4. Thermal stability of carbon nitride nanothreads samples. Thermogravimetric analysis (TGA) of the as-synthesized carbon nitride nanothread sample from 20 to 700°C under argon. Three distinct regions of mass loss, referred to a region A: 30−230 °C (indicated in green), B: 230−330 °C (indicated in blue), and region C: 330−500 °C (indicated in red), can be identified.

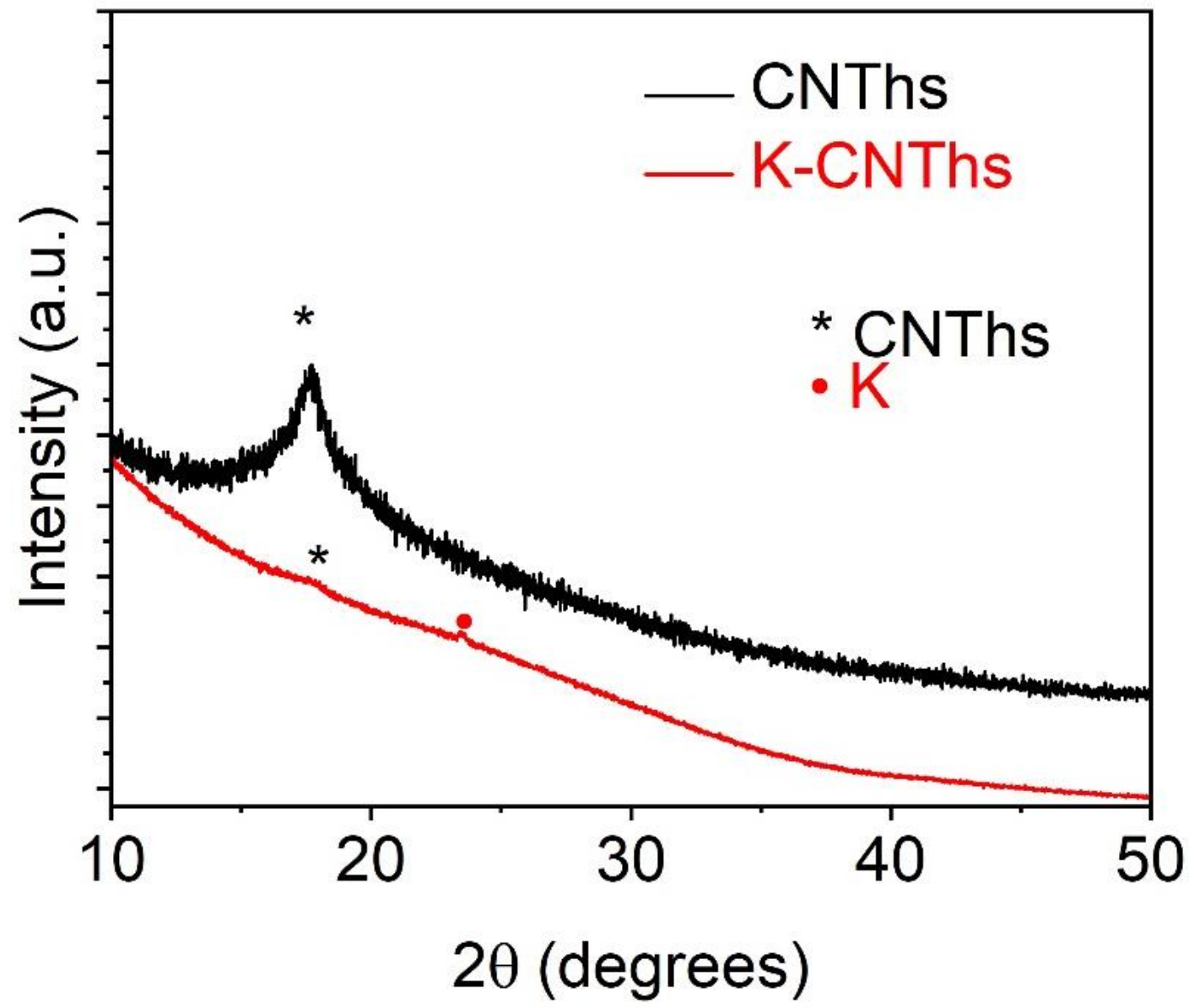

Figure S5. XRD profiles for a sample of furan nanothreads before and after reaction with K. In the pristine nanothreads sample, the (100) broad diffraction peak at 17.64 ˚ corresponds to a d-spacing of 5.0 Å, confirming that the sample contains crystalline furan-derived nanothread bundles. Upon reduction of the nanothreads with K the (100) diffraction peak disappears suggesting that K insertion introduces structural disorder in the 2D pseudohexagonal packed (bundled) lattice.

Due to the bundled nature of carbon nanothreads, potassium metal is expected to infiltrate the sample and confined within the van der Waals spacings between adjacent nanothreads and within the interstitial channels found between the nanothreads. Figure S5 presents XRD profiles for a sample of furan nanothreads before and after reaction with K. In the pristine nanothreads sample, the (100) broad diffraction peak at 17.64 ˚ corresponds to a d-spacing of 5.0 Å, confirming that the sample contains crystalline furan-derived nanothread bundles.[1] Upon reduction of the nanothreads with K the (100) diffraction peak disappears suggesting that K insertion introduces structural disorder in the 2D pseudohexagonal packed (bundled) lattice. The reduction of nanothread bundles yields a product in which potassium atoms intercalate inside the van der Waals spacings between adjacent nanothreads in a bundle in a disordered fashion. This effect has been observed before from XRD studies of SWCNTs upon alkali metal doping.[2,3]

Computation Details

DFT calculations are done to obtain the energy of various structures and the binding energy per potassium atom in different situations. The binding energy per potassium atom $E_{binding\ per\ K}$ is defined as the following

$$E_{threads\ with\ K} = E_{threads} + E_{metal\ per\ K} \cdot N_K + E_{binding\ per\ K} \cdot N_K$$

where N_K is the number of potassium atoms in the structure with potassium intercalated in threads. Negative binding energy would mean favorable for intercalation.

The first set of calculations involves various binding energy of Potassium with isolated non-packed thread. We will show that the types of interaction, from the most intercalation-favorable to the least, are the ionic interaction between N^{-} K^{+} (where the -N- anion is produced by further polymerization of C=N when K is introduced), the electron donation from K to C=N π^*, the coordination between charge-neutral =N- and K, the coordination between K and C=C, and the coordination between K and saturated C and H. The atomic coordinates and energy of this first set of calculations can be found in the folder 'all_isolated' located within the zip file in the Supplemental Information. A summary of this first set of calculations is shown in Table S.

The second set of calculations involves various binding energy of Potassium with packed crystalline threads with different packing arrangements and intercalated geometries. We will identify the preferred packing and intercalated geometry, and recognize that ionic interaction between N^{-} K^{+} are always significantly intercalation-favorable, that the electron donation from K to C=N π^* ranges from intercalation-favorable to neutral depending on the degree of unsaturation and the p-orbital steric repulsion between adjacent C=N. Other types of interactions including the coordination between charge-neutral =N- and K, the coordination between K and C=C, and the

coordination between K and saturated C and H, are always significantly disfavoring for intercalation. The atomic coordinates and energy of this second set of calculations can be found in the folder 'all_packed' located within the zip file in the Supplemental Information. A summary of this second set of calculations is shown in Table S2.

The third and final set of calculations are spin polarized calculations involving the most illustrative and relevant situations selected from the previous two sets of calculations. The structures shown in the main text figure are from this third set. The atomic coordinates and energy of this first set of calculations can be found in the folder ‘best_examples_spin_polarized' located within the zip file in the Supplemental Information. A summary of this third set of calculations is shown in Table S.

All three sets of calculations are done by density functional theory with the PBE functional, and a plane wave basis set with a cutoff of 550 eV as implemented in the VASP software package. Most calculations are done with a reciprocal moment step size finer than 2π/20Å. 4x4x4 k-mesh is used for most structures to achieve this step size, since the shortest lattice parameter is around 5Å. The first two sets of calculations, which are preliminary, are not spin polarized. The third set is spin polarized. The first set of calculations, involving isolated threads, does not use Van der Waals correction. The second and third set of calculation, involving mostly packed threads, use DFT-D3(BJ) Van der Waals correction.

name	energy_eV	dE_to_K0	n_K	binding_E	description
K2_ISPIN1_IVDW0	-2.0616		2		bulk Potassium with 2 K atoms per unit cell
X2_K0_IV-7	-301.735		0		(#unsat_benzene #C=C) isolated anti benzene thread without nitrogen.
X2_K1_IV-7	-301.877	-0.142	1	0.889	
X2_K2_IV-7	-302.87	-1.135	2	0.463	
X2_K0_IV-7_N=__Pi	-283.92		0		(#unsat #C=N) isolated anti thread where C=N π* partially accepting electrons.
X2_K1_IV-7_N=__PiDo	-284.297	-0.377	1	0.654	
X2_K2_IV-7_N=__PiDo	-285.418	-1.498	2	0.282	
X2_K0_IV-7_N-	-280.721		0		(#unsat #C=C, 1) isolated anti thread where N are not in the double bonds and electrons are not accepted. Different Potassium locations are explored here (near C=C or near N).
X2_K1_IV-7_N-	-280.811	-0.091	1	0.94	
X2_K2_IV-7_N-	-281.815	-1.095	2	0.483	
X2_K1_IV-7_N-_KSide	-280.997	-0.277	1	0.754	
X2_K2_IV-7_N-_KSide	-282.074	-1.353	2	0.354	
X2_K0_IV-7_N-asym	-281.199		0		(#unsat #C=C, 2) isolated anti thread where N are not in the double bonds.
X2_K1_IV-7_N-asym	-281.294	-0.096	1	0.935	
X2_K2_IV-7_N-asym	-282.295	-1.097	2	0.483	
X2_K0_IV-12_N=__Pi	-279.291		0		(#unsat #C=N #stack) isolated thread with a stack of C=N.
X2_K2_IV-12_N=__PiDo	-280.788	-1.498	2	0.282	
X2_K0_IV-12_N=__SatManual	-284.068		0		(#sat, #KN-coordination) isolated thread similar to above but the stack of C=N become saturated spontaneously by the added Potassium atoms. Potassium atoms are coordinating with the saturated N.
X2_K1_IV-12_N=__SatStroInt	-284.372	-0.304	1	0.727	
zzX2_K2_IV-12_N=__SatStroInt	-285.381	-1.313	2	0.375	
X2_K0_IV-12_N-__Sat	-284.165		0		(#sat, #KC-coordination) isolated saturated thread where the Potassium atoms are NOT coordinated with the N.
X2_K2_IV-12_N-__SatWeakInt	-285.312	-1.147	2	0.457	
X2_K0_IV-18_N=__Pi	-279.514		0		(#unsat #C=N #stack #some_further_poly #ionic) isolated thread with a stack of C=N that polymerize further, forming bond-order-2 nitrogen and potassium salt. the binding energy and the energy of removing potassium are calculated.
X2_K2_IV-18_N=__Salt22	-283.238	-3.724	2	-0.831	
X2_K2_IV-18_N=__Salt22-ThenRemoveK	-278.982	-4.256	0	-1.097	

Table S1. A summary of the first set of calculations. This set involves the binding energy of potassium on various isolated threads. Only the last group, which has description (#unsat #C=N #stack #some_further_poly #ionic), has negative/intercalation-favorable binding energy with an isolated thread. However, some other groups will become intercalation-favorable in later crystalline calculations when functional groups from multiple threads are coordinating with a single potassium simultaneously from different directions. Group (#unsat #C=C, 1) shows that potassium energetically prefers coordinating with a saturated N over with C=C. The comparison between Group (#unsat #C=C, 1) and Group (#unsat #C=N) shows potassium coordinating with C=N is more favorable for intercalation than C=C or KN coordination. The comparison between Group (#sat, #KC-coordination) and Group (#sat, #KN- coordination) shows potassium prefer to coordinate with saturated N over saturated C and H.

name	raw_E	normalized_E	dE_to_K0	norm_n_K	binding_E	description
K2_ISPIN1_IVDW12	-2.29505	-2.29505		2		bulk Potassium with 2 K atoms per unit cell
12N=_K0	-566.498	-566.498		0		(#unsat #C=N #stack #some_further_poly #ionic) packed threads with stacks of C=N that polyerized further, forming bond-order-2 nitrogen and K salt.
12N=_K2	-569.189	-569.189	-2.691	2	-0.198	
12N=_K4	-575.874	-575.874	-9.376	4	-1.196	
II-3a_Nd_K0	-571.201	-571.201		0		(#unsat #deg-2 #non-rigid) packed degree-2 threads with 4 unsaturated atoms and 2 saturated atoms per precursor
II-3a_Nd_K2	-575.633	-575.633	-4.432	2	-1.069	
II-3a_Nd_K4	-579.099	-579.099	-7.899	4	-0.827	
IV-8_Nd_K0	-575.703	-575.703		0		(#unsat #two-double-bond-squeezed) packed syn-anti thread that has two adjacent double bonds held face-to-face in proximity.
IV-8_Nd_K1	-576.496	-576.496	-0.792	1	0.355	
IV-8_Nd_K2	-578.369	-578.369	-2.666	2	-0.185	
IV-13_Nd_K0	-574.909	-574.909		0		(#unsat, 1) packed threads that are similar to anti which has double bond alternating on different side of the thread.
IV-13_Nd_K2	-577.245	-577.245	-2.336	2	-0.02	
7N=_K0	-575.794	-575.794		0		(#unsat, 2) packed anti threads with different packing geometry was explored
7N=_K2	-577.978	-577.978	-2.184	2	0.055	
7N=_K4	-579.337	-579.337	-3.543	4	0.262	
7N=_K3	-578.804	-578.804	-3.01	3	0.144	
7N=_K2_X2	-1155.96	-577.981	-2.187	2	0.054	
7N=_K3_Z2	-1157.6	-578.802	-3.007	3	0.145	
12StrongInt_K0	-576.381	-576.381		0		(#sat, 1) packed saturated Tube thread that comes from polymerizing the stack of double bonds from thread-12. different packing geometry was explored.
12StrongInt_K2	-577.56	-577.56	-1.179	2	0.558	
12StrongInt_K4	-579.686	-579.686	-3.305	4	0.321	
StrongInt_Quad_K2	-1153.1	-576.549	-0.168	1	0.979	
StrongInt_Tri_K2	-864.957	-576.638	-0.257	1.333	0.955	
12WeakInt_K0	-576.493	-576.493		0		(#sat, 2) packed saturated Tube thread with nitrogen atoms in different position from ones above.
12WeakInt_K2	-578.063	-578.063	-1.57	2	0.362	
12WeakInt_K4	-580.012	-580.012	-3.519	4	0.268	
12WeakInt_K3	-578.719	-578.719	-2.227	3	0.405	
IV-15_Nd_K0	-859.187	-859.187		0		(#unsat #chiral) packed chiral threads that has more randomized coordination pattern with potassium.
IV-15_Nd_K4	-861.198	-861.198	-2.011	4	0.645	
IV-15_Nd_K8	-864.642	-864.642	-5.454	8	0.466	

Table S2. A summary of the second set of calculations. This set involves the binding energy of potassium on various packed crystalline threads. The first three groups, which have description (#unsat #C=N #stack #some_further_poly #ionic), (#unsat #deg-2 #non-rigid), and (#unsat #two-double-bond-squeezed) have significantly negative/intercalation-favorable binding energy. The next two groups, (#unsat, 1) and (#unsat, 2) have near-zero binding energy. Saturated threads in groups (#sat, 1) and (#sat, 2) all have significantly positive/intercalation-unfavorable binding energy.

name	energy	dE_to_K0	n_K	binding_E	description	previous_name	folder
00_bulk-potassium_K2	-2.287		2		Bulk Potassium with 2 K atoms per unit cell		
01_syn-anti_K0	-575.701		0		Packed syn-anti threads that have two adjacent double bonds held face-to-face in proximity.	IV-8_Nd_K0	packed
01_syn-anti_K2	-578.494	-2.793	2	-0.253		IV-8_Nd_K2	packed
02_anti_K0	-575.798		0		Packed anti threads	7N=_K0	packed
02_anti_K2	-578.006	-2.208	2	0.040		7N=_K2	packed
03_pseudo-anti_K0	-574.923		0		Packed threads similar to anti, which has C=N on alternating side of each thread.	IV-13_Nd_K0	packed
03_pseudo-anti_K2	-577.308	-2.385	2	-0.049		IV-13_Nd_K2	packed
04_degree-2_K0	-571.185		0		Packed degree-2 threads with 4 unsaturated atoms and 2 saturated atoms per precursor	II-3a_Nd_K0	packed
04_degree-2_K2	-575.636	-4.451	2	-1.082		II-3a_Nd_K2	packed
04_degree-2_K4	-579.495	-8.310	4	-0.934		II-3a_Nd_K4	packed
05_stack-1_isolated_K0_pre-cata	-281.886		0		Isolated thread with a stack of C=N that polyerized further, forming bond-order-2 nitrogen and potassium salt. The binding energy and the energy of removing potassium are calculated.	X2_K0_IV-18_N=__Pi	isolated
05_stack-1-isolated_K2_post-cata	-285.870	-3.984	2	-0.848		X2_K2_IV-18_N=__Salt22	isolated
05_stack-1-isolated_K0_post-cata-remove-K	-281.924	-3.946	0	-0.829		X2_K2_IV-18_N=__Salt22-ThenRemoveK	isolated
06_stack-2_K0_pre-catalysis	-566.662		0		Packed thread with a stack of C=N that polyerized further, forming bond-order-2 nitrogen and potassium salt. The binding energy and the energy of removing potassium are calculated.	12N=_K0	packed
06_stack-2_K2_post-catalysis	-576.110	-9.448	2	-3.580		12N=_K2	packed
06_stack-2_K4_post-catalysis	-579.896	-13.233	4	-2.165		12N=_K4	packed
11_saturated_K0_weak-interaction	-576.493		0		Packed saturated Tube thread that comes from polymerizing the stack of double bonds from thread-12. The binding energy is unfavorable for intercalation.	12StrongInt_K0	packed
11_saturated_K2_weak-interaction	-578.083	-1.590	2	0.349		12StrongInt_K2	packed
11_saturated_K4_weak-interaction	-580.044	-3.551	4	0.256		12StrongInt_K4	packed
12_anti-no-N-in-alkene_K0_weak-interaction	-283.220		0		Isolated saturated Tube thread that comes from polymerizing the stack of double bonds from thread-12. The binding energy is unfavorable for intercalation.	X2_K0_IV-7_N-	isolated
12_anti-no-N-in-alkene_K2_CCK-weak-interaction	-284.422	-1.203	2	0.542		X2_K2_IV-7_N-	isolated
12_anti-no-N-in-alkene_K2_NK-weak-interaction	-284.857	-1.637	2	0.325		X2_K2_IV-7_N-_KSide	isolated

Table S3. A summary of the third set of calculations. This set involves structures that best illustrate different types of thread potassium interaction and their binding energy. These structures are selected from the previous two sets of calculations. Their previous names in the previous two sets of calculations are shown in the previous_name and folder columns.

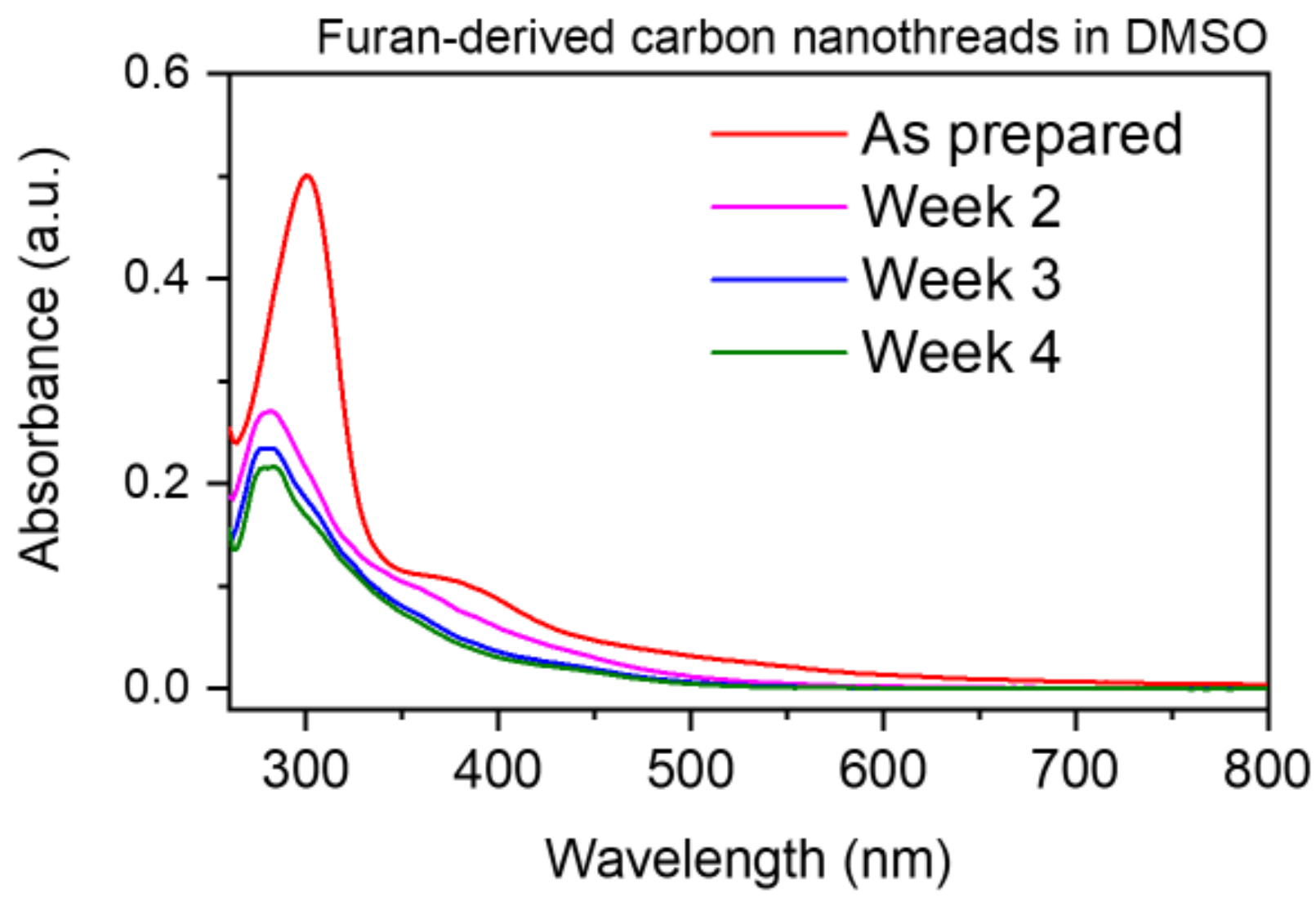

Figure S6. Evolution of the UV–vis absorption spectra of furan-derived carbon nanothread dispersions in DMSO. The as-prepared dispersion shows a strong absorption peak at ~300 nm, attributed to charged carbon species. Upon exposure to air, this peak decreases in intensity and blue shifts to ~280 nm, reflecting the discharge or neutralization of the reduced nanothreads. The spectra remain stable thereafter, indicating that the nanothreads remain well dispersed even after air exposure and loss of charge.

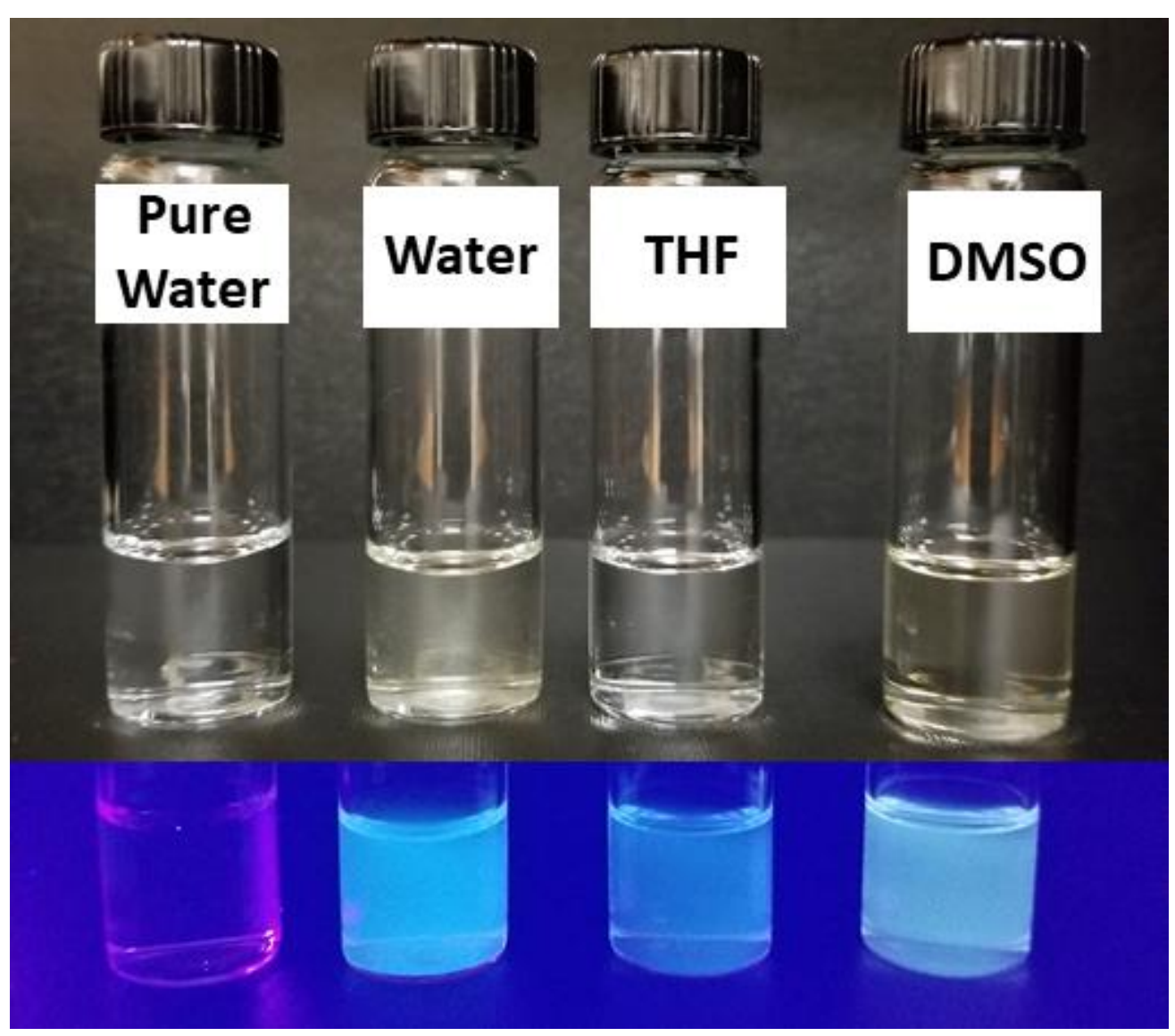

Figure S7. Photograph showing that dilute suspensions of carbon nitride nanothreads dispersed in different solvents are transparent and display strong blue fluorescence when illuminated with a 365 nm UV lamp.

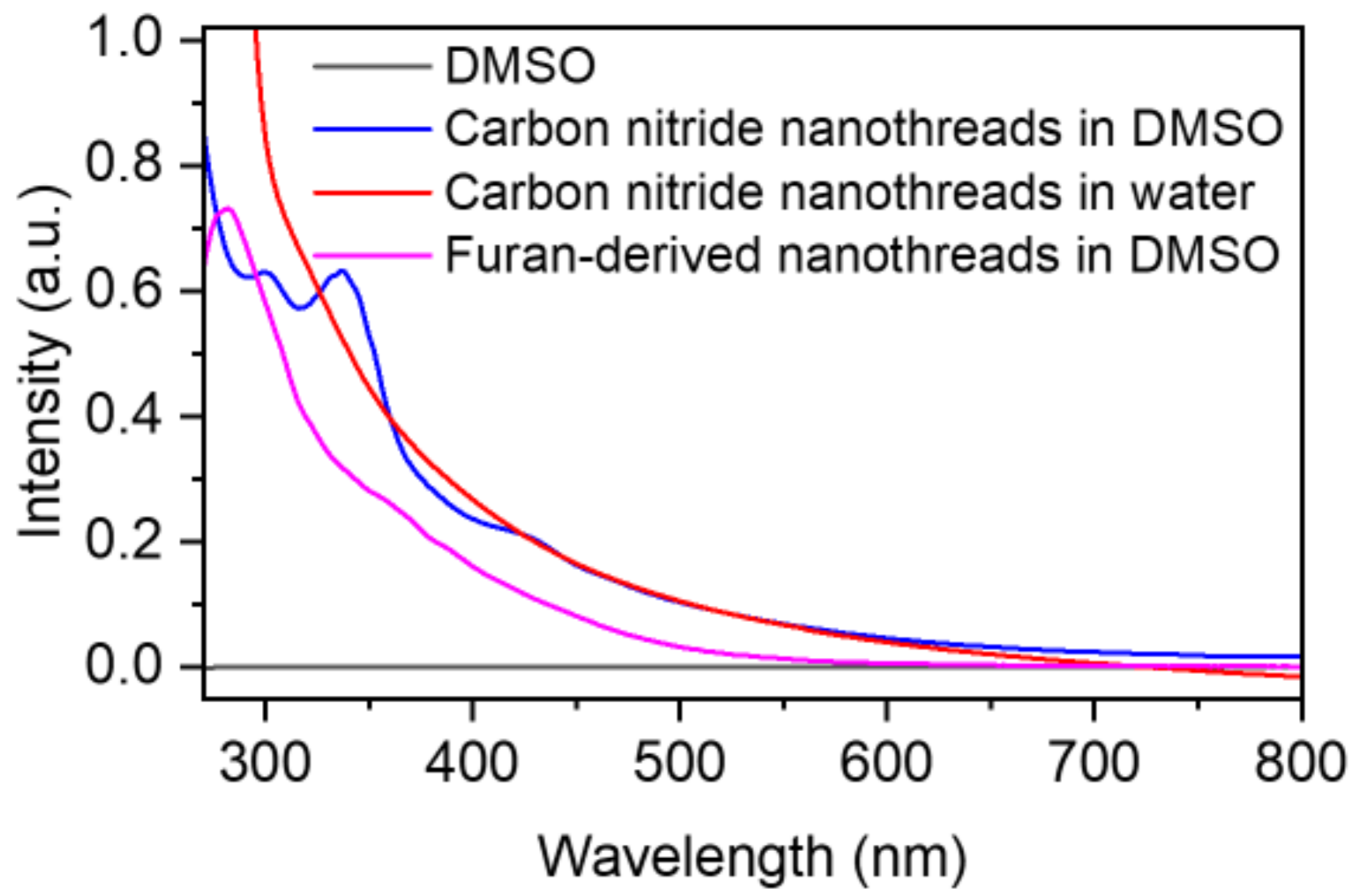

Figure S8. (a) UV–vis absorption spectra of a carbon nitride nanothread dispersion in DMSO and in water compared with that of furan-derived nanothreads, showing distinct optical behaviors. The pyridine-derived nanothreads exhibit a red-shifted absorption edge relative to the furan-derived nanothreads, consistent with their predicted smaller band gap arising from electron rich nitrogen incorporation.

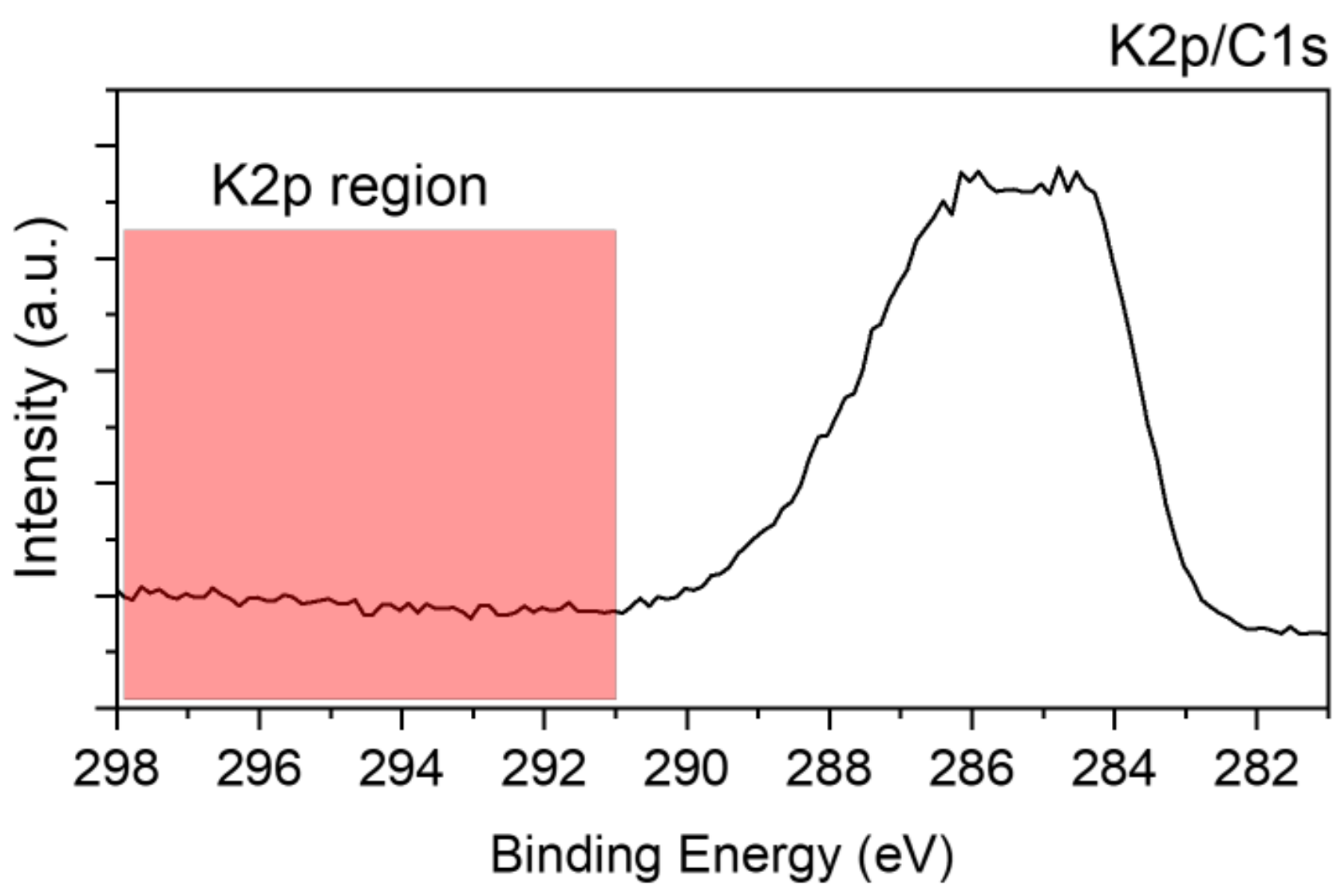

Figure S9. XPS spectrum of the K 2p and C 1s regions of the carbon nitride nanothread films after transfer to a silicon oxide substrate, showing no detectable K 2p signal. The absence of potassium peaks confirms that K^+ ions are fully removed during the centrifugation and washing steps, yielding clean films composed solely of carbon nitride nanothreads deposited on the substrate. This result indicates that potassium acts only as a charge-transfer agent during the intercalation–exfoliation process and does not remain in the final material.

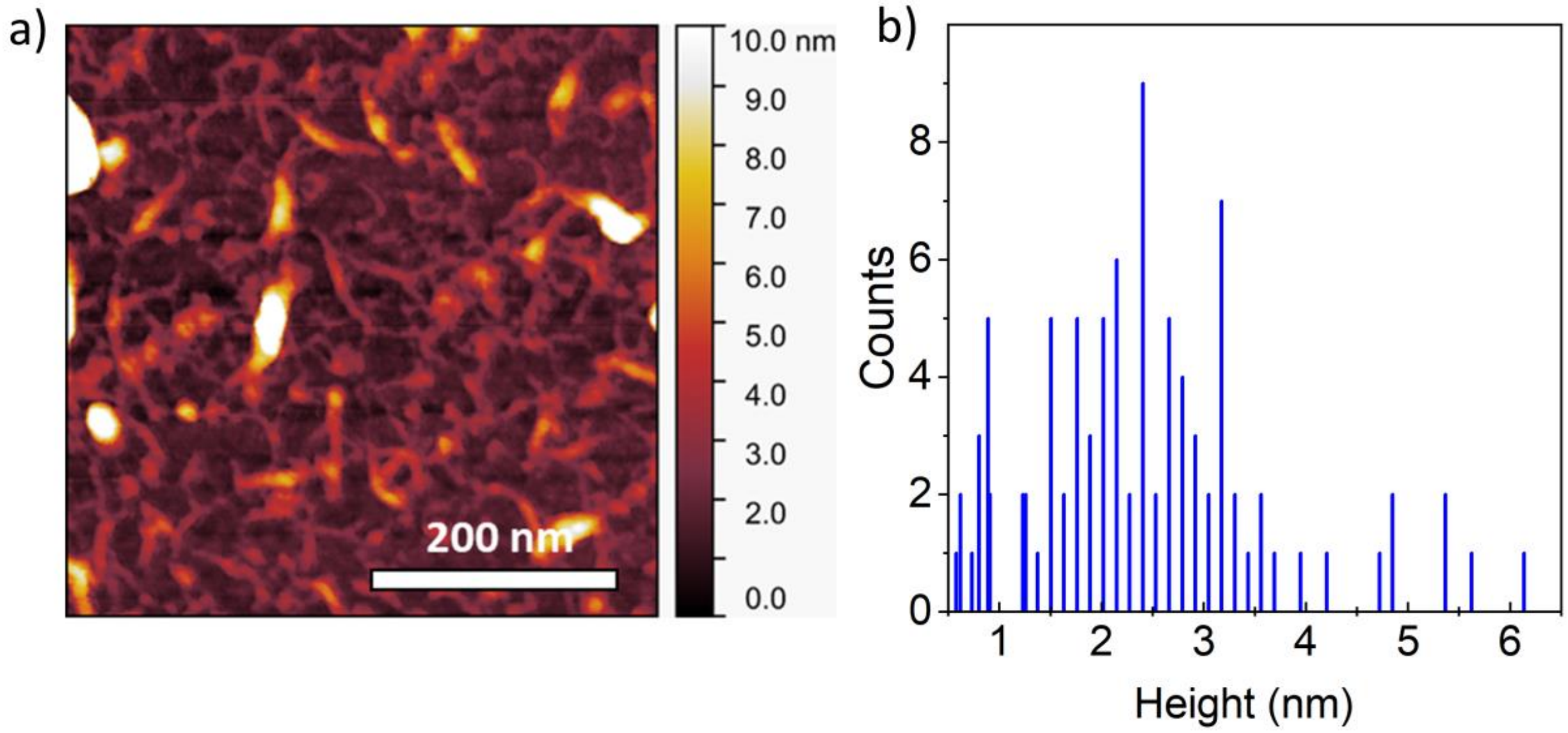

Figure S10. (a) Determination of length of individualized carbon nitride nanothreads. (b) Statistical distribution analysis of the AFM image of the carbon nitride nanothread film depicted in Figure 4c. The majority of the nanothreads in the thin film are individualized < 2 nm, while some are observed as small bundles consisting of 2-5 nanothreads < 4 nm.

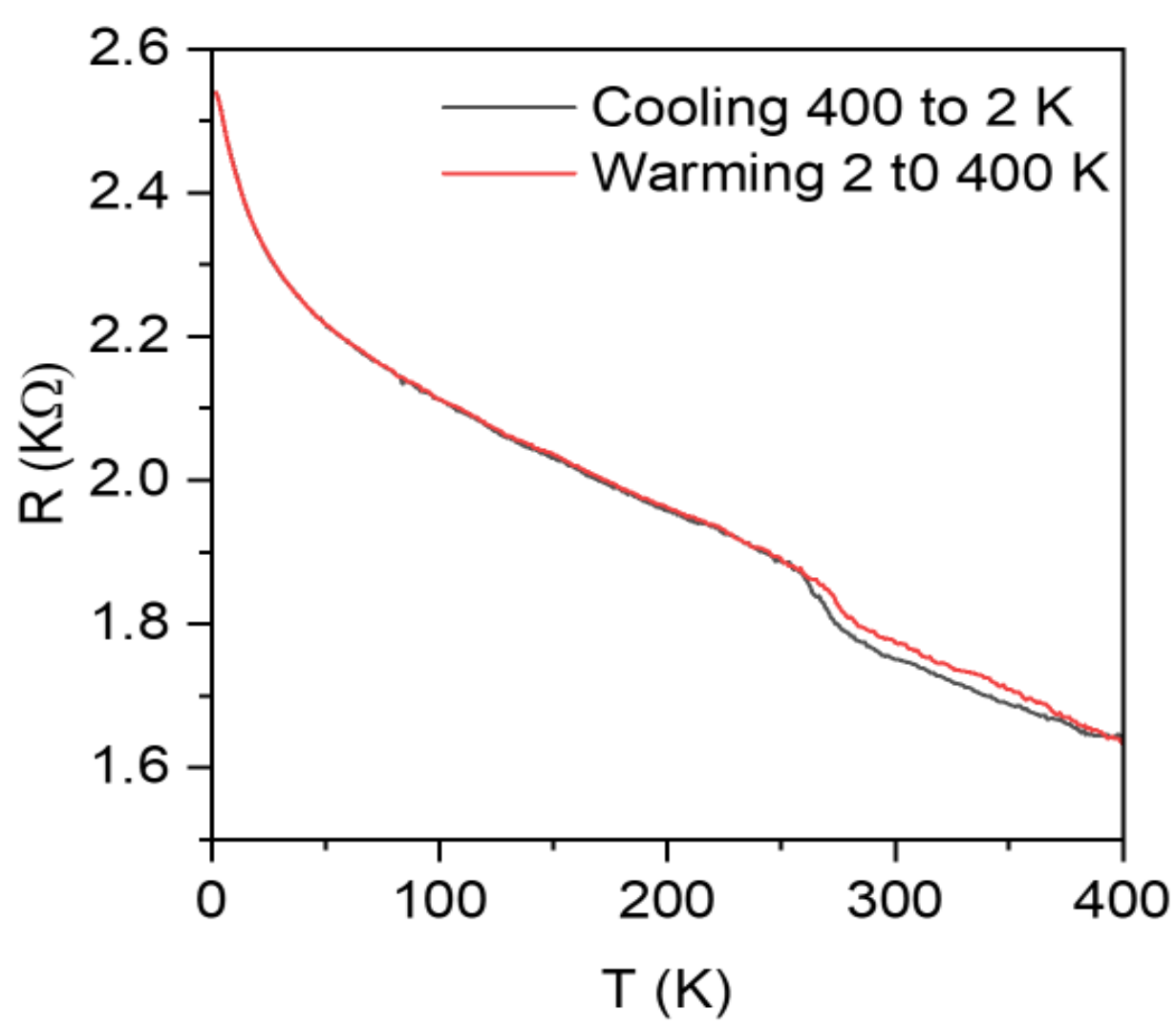

Figure S11. Temperature-dependent resistance (R) of the carbon nitride nanothread film measured during a thermal cycle from 400 K to 2 K (cooling, black curve) and back to 400 K (warming, red curve). A small hysteresis is observed between the cooling and warming curves, particularly near 250 K, indicating possible thermally activated structural or interfacial changes.

References

[1] Huss S, Wu S, Chen B, et al. Scalable Synthesis of Crystalline One-Dimensional Carbon Nanothreads through Modest-Pressure Polymerization of Furan. ACS Nano. 2021;15(3):4134–4143.

[2] Claye AS, Nemes NM, Jánossy A, et al. Structure and electronic properties of potassium-doped single-wall carbon nanotubes. Phys Rev B. 2000;62(8):R4845–R4848.

[3] Claye AS, Fischer JE, Huffman CB, et al. Solid-State Electrochemistry of the Li Single Wall Carbon Nanotube System. J Electrochem Soc. 2000;147(8):2845.
